# X chromosome associations with chronic obstructive pulmonary disease and related phenotypes: an X chromosome-wide association study

**DOI:** 10.1186/s12931-023-02337-1

**Published:** 2023-02-01

**Authors:** Lystra P. Hayden, Brian D. Hobbs, Robert Busch, Michael H. Cho, Ming Liu, Camila M. Lopes-Ramos, David A. Lomas, Per Bakke, Amund Gulsvik, Edwin K. Silverman, James D. Crapo, Terri H. Beaty, Nan M. Laird, Christoph Lange, Dawn L. DeMeo

**Affiliations:** 1 Division of Pulmonary Medicine, Boston Children’s Hospital, Harvard Medical School, Boston, MA USA; 2 Channing Division of Network Medicine, Brigham and Women’s Hospital, Harvard Medical School, 181 Longwood Ave, Boston, MA 02115 USA; 3 Division of Pulmonary and Critical Care Medicine, Brigham and Women’s Hospital, Harvard Medical School, Boston, MA USA; 4 Division of Pulmonology, Allergy, and Critical Care, U.S. Food and Drug Administration, Silver Spring, MD USA; 5 Bioinformatics and Computational Biology Program, Worcester Polytechnic Institute, Worcester, MA USA; 6 Department of Biostatistics, Harvard T.H. Chan School of Public Health, Boston, MA USA; 7 UCL Respiratory, University College London, London, UK; 8 Department of Clinical Science, University of Bergen, Bergen, Norway; 9 Division of Pulmonary Sciences and Critical Care Medicine, National Jewish Health, Denver, CO USA; 10 Johns Hopkins School of Public Health, Baltimore, MD USA

**Keywords:** COPD, Lung function, Emphysema, X chromosome-wide association study, Sex differences, X chromosome inactivation

## Abstract

**Background:**

The association between genetic variants on the X chromosome to risk of COPD has not been fully explored. We hypothesize that the X chromosome harbors variants important in determining risk of COPD related phenotypes and may drive sex differences in COPD manifestations.

**Methods:**

Using X chromosome data from three COPD-enriched cohorts of adult smokers, we performed X chromosome specific quality control, imputation, and testing for association with COPD case–control status, lung function, and quantitative emphysema. Analyses were performed among all subjects, then stratified by sex, and subsequently combined in meta-analyses.

**Results:**

Among 10,193 subjects of non-Hispanic white or European ancestry, a variant near *TMSB4X,* rs5979771, reached genome-wide significance for association with lung function measured by FEV_1_/FVC ($\beta$ 0.020, *SE* 0.004, *p* 4.97 × 10^–08^), with suggestive evidence of association with FEV_1_ ($\beta$ 0.092, *SE* 0.018, *p* 3.40 × 10^–07^). Sex-stratified analyses revealed X chromosome variants that were differentially trending in one sex, with significantly different effect sizes or directions.

**Conclusions:**

This investigation identified loci influencing lung function, COPD, and emphysema in a comprehensive genetic association meta-analysis of X chromosome genetic markers from multiple COPD-related datasets. Sex differences play an important role in the pathobiology of complex lung disease, including X chromosome variants that demonstrate differential effects by sex and variants that may be relevant through escape from X chromosome inactivation. Comprehensive interrogation of the X chromosome to better understand genetic control of COPD and lung function is important to further understanding of disease pathology.

*Trial registration* Genetic Epidemiology of COPD Study (COPDGene) is registered at ClinicalTrials.gov, NCT00608764 (Active since January 28, 2008). Evaluation of COPD Longitudinally to Identify Predictive Surrogate Endpoints Study (ECLIPSE), GlaxoSmithKline study code SCO104960, is registered at ClinicalTrials.gov, NCT00292552 (Active since February 16, 2006). Genetics of COPD in Norway Study (GenKOLS) holds GlaxoSmithKline study code RES11080, Genetics of Chronic Obstructive Lung Disease.

**Supplementary Information:**

The online version contains supplementary material available at 10.1186/s12931-023-02337-1.

## Background

Chronic obstructive pulmonary disease (COPD) is a disease with both environmental and genetic risk factors [1, 2]. Several genome-wide association studies (GWAS) have demonstrated multiple autosomal associations [3]. However, much of the overall heritability of COPD remains unexplained [4]. One potential source of additional information is genetic variation on the X chromosome, which has been excluded in most prior COPD GWAS [5].

Inclusion of the X chromosome in association studies requires modified quality control procedures and poses statistical challenges distinct from autosomes, including the presence of only one X chromosome in males and random inactivation of one X in females [5, 6]. Standard GWAS methodology for autosomal variants relies on the fact that each locus has 2 alleles and thus each paired autosomal variant generally has three possible genotypes, 0/1/2. If these methods are applied to the X chromosome, where males are hemizygous, the X variants would be coded as 0/1 for males and 0/1/2 for females. This may limit the ability to detect associations compared to methods that consider male X chromosome genetic variants as 0/2 [7]. Underutilization of genetic information on the X chromosome has been noted along with calls for greater incorporation of allosomal data in future GWAS of complex human traits and diseases [8].

The motivation of this study is to properly include and assess the X chromosome variation on a genome-wide level to more completely understand sex differences in COPD. Women are more susceptible to severe, early-onset COPD [9]. Disease features such as emphysema and exacerbations show sex differences, as do disease manifestations of dyspnea, depression, and anxiety [10, 11]. Several GWAS have begun incorporating the X chromosome [12–14]. Variants associated with lung function on the X chromosome have been identified in previous GWAS [14–16]. There has been limited prior interrogation of the X chromosome in GWAS of COPD, and related phenotypes including emphysema, that employed comprehensive accounting for the X chromosome and included sex-stratified analysis [16, 17]. Prior studies have shown the significance of sex differences and the X chromosome in cardiovascular disease and related traits, but similar examinations have not been performed in COPD [18].

This study aims to perform an X chromosome-wide association study (XWAS) and meta-analyses of COPD datasets, including sex-stratified analyses to test for association between genetic loci on the X chromosome with COPD and related phenotypes of lung function and quantitative emphysema (emphysema) on chest computed tomography (CT) scans. To achieve this, we utilize quality control and statistical methods that specifically consider X chromosome inheritance patterns and inactivation. We hypothesize that the X chromosome harbors variants important in determining risk of COPD and related quantitative phenotypes, and that X chromosome variants may drive some sex differences in COPD manifestations.

Some results have been previously reported as an abstract [19].

## Methods

### Study participants and phenotyping

Study participants were current and former smokers in three previously described studies: Genetic Epidemiology of COPD Study (COPDGene, non-Hispanic white subset), Genetics of COPD in Norway Study (GenKOLS), and Evaluation of COPD Longitudinally to Identify Predictive Surrogate Endpoints Study (ECLIPSE) [1, 20, 21]. Subjects with COPD (cases) were defined by Global Initiative for Chronic Obstructive Lung Disease (GOLD) airflow limitation severity grades 2–4, on the basis of standardized post-bronchodilator spirometry with Forced Expiratory Volume in one second (FEV_1_) < 80% predicted, and FEV_1_ to Forced Vital Capacity (FVC) ratio < 0.7 [22]. Control subjects (controls) included smokers with normal spirometry (FEV_1_ ≥ 80%, FEV_1_/FVC ≥ 0.7). Measurement of quantitative emphysema (emphysema) was defined as the natural logarithm transform of the percentage of lung voxels with density less than − 950 Hounsfield units on chest CT inspiratory images (log − 950), determined using Thirona software (http://www.thirona.eu) for COPDGene, and Slicer software (http://www.slicer.org) for ECLIPSE and GenKOLs [23].

Study subjects provided written informed consent and each study's research protocol was approved by institutional review boards at participating institutions. Study phenotypes are further defined in the supplement (Additional file 1). Datasets from COPDGene (accession number phs000179.v6.p2) and ECLIPSE (accession number phs001252.v1.p1) are publicly available in dbGaP.

### Genotyping, quality control, and imputation

Genotyping for all studies was performed using various Illumina (San Diego, CA) platforms. Genotyping and initial quality control methods have been previously described, and included cleaning based on subject-level missingness, cryptic relatedness, and sex checks based on X and Y chromosomes [21, 24]. Principal components of genetic ancestry were generated separately, based on genotyped autosomal data in each case–control population using EIGENSOFT, as previously described. The pseudoautosomal region was excluded from the X chromosome prior to association analysis, defined for human genome build 19 (GRCh37/hg19, https://www.ncbi.nlm.nih.gov/grc/human) by X chromosome base pair coordinates 60,001–2,699,520 and 154,931,044–155,260,560.

**Fig. 1:**
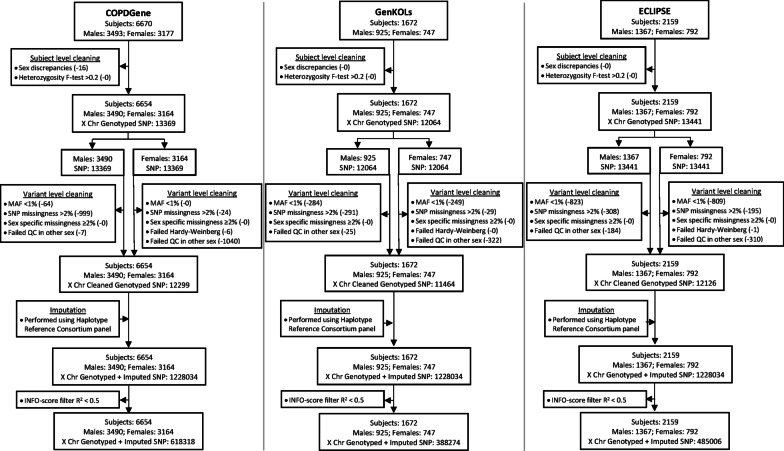
Cleaning and imputation by study. Steps in subject cleaning, variant cleaning, and imputation prior to X chromosome association analysis. Numbers in parentheses represent subjects or variants removed. Abbreviations: *Chr* chromosome, *SNP* single nucleotide polymorphism, *MAF* minor allele frequency, *QC* quality control

Additional quality control steps for variants on the X chromosome followed published recommendations and were performed using PLINK v1.9 (Fig. 1) [5, 25]. A full description of study based subject level data cleaning as well as imputation methods are in the supplement (Additional file 1).

### Statistical analysis

X chromosome variants were tested in each of the three studies independently for association with four individual phenotypes: COPD case–control status, FEV_1_ in liters, FEV_1_/FVC and quantitative emphysema using logistic regression for COPD status and linear regression analysis for quantitative phenotypes. XWAS were run in all subjects and additionally stratified by sex as demonstrated in Additional file 1: Figure S1a–c for COPDGene. Regression models for each phenotype were adjusted for age, pack-years of smoking, and principal components of genetic ancestry for all analyses [6, 25, 26]. For FEV_1_ in liters height was included as a covariate. For the analyses of all subjects, sex was considered as a covariate (females 0, males 1). For the emphysema analyses, additional covariates included current smoking, body mass index, and scanner model if more than one scanner was used in that study. Variants were excluded if they had a minimum minor allele frequency of < 0.01, were multiallelic, or in the COPD case/control XWAS if they were present in only one group.

Clayton’s method for XWAS was employed, which accounts for both female X chromosome inactivation (XCI) and male X chromosome hemizygosity [6, 27]. Males carry only one copy of the X chromosome, while in female most loci are subject to XCI so that a female will have approximately half of their cells with one copy active and the remainder will have the other copy activated. Males are equivalent to homozygous females in respect to such loci. Clayton’s method for analysis of the X chromosome, implemented in PLINK v2.0, performs logistic regression models with one degree of freedom encoding female loci as 0 (homozygous for reference allele), 1 (heterozygous), or 2 (homozygous for alternate allele) and male loci as 0 (no copies of alternate allele) or 2 (single copy of alternate allele). With this approach the heterozygous female genotype falls between two homozygous genotypes on the linear predictor scale, thus accounting for XCI as only 50% of cells will have a normal active allele [27].

Results were subsequently combined into fixed-effects meta-analyses using all quality-controlled variants from COPDGene, ECLIPSE and GenKOLs studies with PLINK v1.9 (Additional file 1: Figure S2). Meta-analyses were run for each of the four phenotypes, both for all subjects and separately in sex-stratified datasets. Variants were excluded if only present in one study. Testing for sex-difference was performed comparing effect estimates among males and females among the top suggested associations in the sex-stratified meta-analysis, with sex-difference significance assessed at *P* < 0.05 [28]. A description of methods for significance thresholds, the suggestive level of association examined, and annotation is in the supplement (Additional file 1).

### Replication

Ten X chromosome variants previously discovered in GWAS for lung function were examined for replication in the meta-analysis results for this XWAS [14, 16, 17]. One variant, rs28382751 from Shrine et al. was not evaluated as it was not present in our study.

## Results

### Subjects

A total of 10,193 subjects of non-Hispanic white or European ancestry with X chromosome data were included in the analysis. Baseline characteristics and summary statistics for each dataset among all subjects and in sex-stratified subsets are shown in Table 1. The largest study population was from COPDGene, with 6631 subjects, while GenKOLs had 1658 subjects and ECLIPSE 1904 subjects. Compared to the other two studies, GenKOLs had fewer mean pack-years of smoking history, and a higher proportion of current smokers. Compared to the other two studies, ECLIPSE, which enrolled a majority of subjects with COPD and comparatively few control smokers, had fewer female subjects and more severe disease. Among males and females in each study group, female subjects were slightly younger, had a shorter pack-year smoking history, and had less severe disease, characterized by higher lung function, less emphysema (especially among females in ECLIPSE), and a lower proportion of subjects with COPD.

**Table 1:** Characteristics of study populations

	COPDGene			GenKOLS			ECLIPSE		
	All Subjects	Males	Females	All Subjects	Males	Females	All Subjects	Males	Females
Subjects with spirometry	6631	3474 (52.39%)	3157 (47.61%)	1658	917 (55.31%)	741 (44.69%)	1904	1258 (66.07%)	646 (33.93%)
Mean age, years (SD)	62.03 (8.84)	62.38 (8.81)	61.65 (8.85)	60.69 (11.05)	61.88 (11.15)	59.21 (10.74)	63.14 (7.51)	63.62 (7.43)	62.22 (7.59)
Mean pack Year History (SD)	47.27 (26.03)	51.54 (28.06)	42.57 (22.68)	25.98 (17.41)	29.49 (18.86)	21.64 (14.28)	48.7 (27.75)	52.12 (29.66)	42.14 (22.20)
Current smoking (%)	2594 (39.12%)	1385 (39.87%)	1209 (38.30%)	731 (44.09%)	377 (41.11%)	354 (47.77%)	661 (35.46%)*	422 (34.20%)*	239 (37.94%)*
GOLD II-IV COPD (%)	2803 (42.27%)	1564 (45.02%)	1239 (39.25%)	853 (51.45%)	513 (55.94%)	340 (45.88%)	1726 (91.08%)	1155 (92.33%)	571 (88.66%)
FEV_1,_ % predicted (SD)	73.56 (25.90)	72.41 (26.49)	74.83 (25.18)	72.18 (26.17)	70.06 (26.19)	74.80 (25.92)	53.13 (23.23)	51.33 (22.74)	56.64 (23.78)
FEV_1_, L (SD)	2.22 (0.95)	2.51 (1.03)	1.88 (0.72)	2.38 (1.10)	2.66 (1.19)	2.04 (0.87)	1.51 (0.78)	1.63 (0.83)	1.28 (0.63)
FEV_1_/FVC (SD)	64.09 (16.61)	62.59 (16.93)	65.74 (16.09)	64.84 (16.92)	63.15 (17.24)	66.94 (16.28)	47.75 (14.90)	46.57 (14.77)	50.04 (14.89)
Subjects with chest CT data	6245	3267	2978	827	482	345	1502	975	527
Emphysema, log -950 (SD)	0.94 (1.71)	1.26 (1.54)	0.60 (1.82)	0.56 (1.80)	0.91 (1.66)	0.07 (1.87)	2.42 (1.11)	2.50 (1.05)	2.29 (1.21)

Comparison of characteristics of subjects from each the three study populations among all study participant and by sex. *Data is missing for some subjects. Abbreviations: *SD* standard deviation; *GOLD* Global Initiative for Chronic Obstructive Lung Disease; *COPD* chronic obstructive pulmonary disease; *FEV1* Forced expiratory volume in one second; *L* liters; *FVC* forced vital capacity; *CT* computed tomography

Subject level cleaning, variant level cleaning, and imputation of genotyped data was done individually for each study (Fig. 1). The resultant variants were included in the XWAS, with 618318 genotyped and imputed variants in COPDGene, 388274 in GenKOLs, and 485006 in ECLIPSE. XWAS were performed separately for each phenotype among all subjects and then in sex-stratified analyses (Additional file 1: Figure S1a–c demonstrates the XWAS analyses for COPDGene). This included association studies for each of the four phenotypes assessed separately in each of the three study strata: all subjects, males, and females. Results were combined using fixed-effects meta-analyses, one for each of the four phenotypes (Additional file 1: Figure S2), with 223295–224268 variants included in each meta-analysis depending on phenotype and population.

### XWAS

**Fig. 2:**
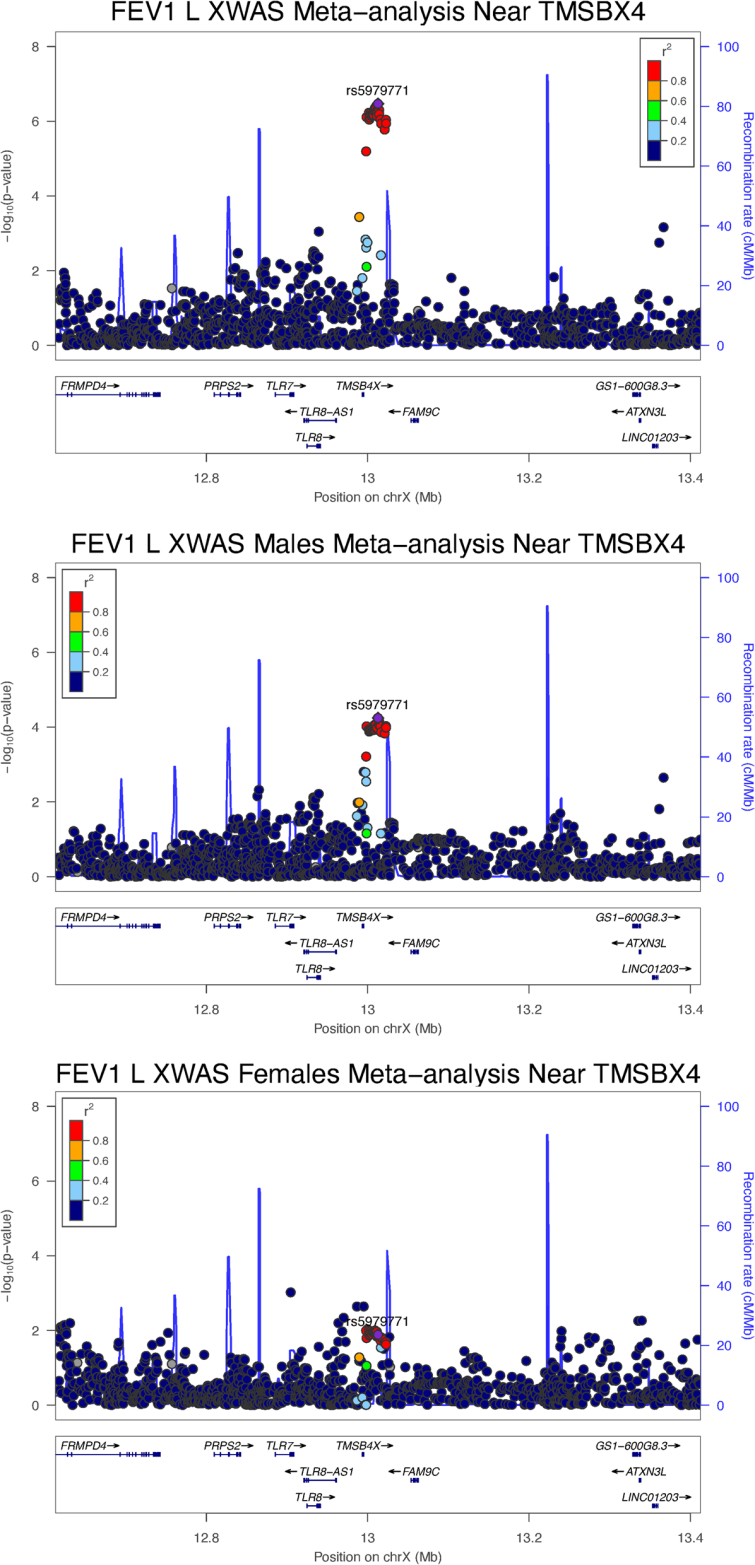
Meta-analysis locus plots. Plots for rs5979771, the genome-wide association for FEV_1_ near TMSB4 X in the XWAS meta-analysis among all subjects and in sex-stratified populations. Abbreviations: *COPD* chronic obstructive pulmonary disease, *XWAS* X chromosome association study, *FEV*_*1*_ forced expiratory volume in one second, *L* liters, *FVC* forced vital capacity

Top suggestive associations from the all-subjects and sex-stratified XWAS meta-analyses are presented in Table 2 and Additional file 2: Table S1. Figure 2 and Additional file 1: Figure S3 provide locus plot visualizations of selected top association results. Quantile–Quantile and Manhattan plots for the meta-analyses are shown in Additional file 1: Figure S4. The top association in rs5979771, a variant closest to *TMSB4X,* achieved genome-wide significance for association with FEV_1_/FVC among all subjects (Beta ($\beta )$ 0.020, standard error (*SE)* 0.004, *p* 4.97 × 10^–08^). The same variant was also the top suggestive variant to show evidence of association with FEV_1_ among all subjects. No other variants reached genome-wide significance, but suggestive associations approaching genome-wide significance were identified. Power calculations can be found in Additional file 1.

**Table 2:** XWAS Meta-analysis Top Suggested Associations in COPD Related Phenotypes

Phenotype	SNP	EA/Other	EAF	Studies	All Subjects			Males			Females			Sex Difference Pvalue			
					OR	95% CI	P value	OR	95% CI	P value	OR	95% CI	P value			Annotated gene	Locus
COPD	**rs138704174**	**A/G**	**0.03**	**3**	**1.58**	**(1.31,1.91)**	**3.55E-06**	1.47	(1.16,1.87)	1.54E-03	1.78	(1.29,2.45)	4.32E-04	3.48E-01		***SH3KBP1***^‡^	**Xp22.12**
	**rs5928132**	**T/C**	**0.39**	**3**	0.89	(0.84,0.95)	2.19E-04	**0.85**	**(0.79,0.91)**	**9.03E-06**	0.99	(0.90,1.10)	8.97E-01	1.30E-02	*	***DMD***^***‡***^	**Xp21.1**
	rs145916556	A/G	0.10	3	0.90	(0.81,1.00)	4.52E-02	1.02	(0.91,1.15)	7.04E-01	0.65	(0.53,0.78)	7.61E-06	4.84E-05	*	*LINC02595*	Xp11.3
	**rs150086151**	**T/A**	**0.01**	**3**	1.55	(1.14,2.12)	5.45E-03	0.87	(0.60,1.25)	4.52E-01	**3.83**	**(2.20,6.66)**	**2.65E-06**	1.18E-05	*	***LOC112268302***^‡^	**Xq24**

					Beta	SE	P value	Beta	SE	P value	Beta	SE	P value				
FEV_1_, L	**rs5979771**	**C/T**	**0.05**	**3**	**0.092**	**0.018**	**3.40E-07**	0.095	0.023	5.70E-05	0.070	0.028	1.30E-02	4.91E-01		***TMSB4X***	**Xp22.2**
	**rs5927319**	**G/A**	**0.25**	**3**	− 0.024	0.009	1.07E-02	− 0.012	0.012	3.33E-01	− **0.063**	**0.014**	**9.84E-06**	6.43E-03	*	***TMEM47***	**Xp21.1**
	rs5960415	T/C	0.04	3	− 0.101	0.022	6.55E-06	− 0.099	0.029	5.60E-04	− 0.100	0.037	6.58E-03	9.80E-01		*PAGE5*	Xp11.21
	rs2375465	T/C	0.03	3	− 0.105	0.023	7.73E-06	− 0.105	0.030	4.79E-04	− 0.102	0.039	9.69E-03	9.44E-01		*FOXR2*^§^	Xp11.21
	rs139674496	C/T	0.03	3	− 0.105	0.023	5.59E-06	− 0.102	0.030	5.81E-04	− 0.114	0.039	3.40E-03	8.13E-01		*RRAGB*	Xp11.21
	**rs5965560**	**G/A**	**0.01**	**2**	0.154	0.036	1.93E-05	**0.244**	**0.049**	**8.52E-07**	− 0.047	0.055	3.99E-01	8.64E-05	*	***OPHN1***^‡^	**Xq12**
	rs142755000	A/G	0.03	3	− 0.134	0.029	4.30E-06	− 0.149	0.040	1.91E-04	− 0.105	0.041	1.17E-02	4.40E-01		*HMGN5*	Xq21.1
	rs185387095	T/A	0.01	2	− 0.187	0.044	2.84E-05	− 0.311	0.067	4.29E-06	− 0.118	0.060	4.79E-02	3.30E-02	*	*HMGN5*^*‡*^	Xq21.1
	rs145089551	A/G	0.01	2	− 0.164	0.042	8.62E-05	− 0.310	0.063	1.16E-06	− 0.052	0.054	3.42E-01	2.01E-03	*	*SH3BGRL*	Xq21.1
	rs6616818	C/G	0.48	3	0.038	0.008	3.85E-06	0.048	0.011	9.66E-06	0.007	0.012	5.93E-01	1.17E-02	*	*POU3F4*	Xq21.1
	rs185667977	G/T	0.01	2	0.287	0.059	1.66E-06	0.307	0.071	1.76E-05	–	–	–	–		*FMR1*^‡^	Xq27.3
FEV_1_/FVC	**rs5979771**	**C/T**	**0.05**	**3**	**0.020**	**0.004**	**4.97E-08**	0.021	0.004	2.57E-06	0.019	0.007	6.58E-03	8.57E-01		***TMSB4X***	**Xp22.2**
	rs12015039	A/G	0.06	3	− 0.011	0.004	2.01E-03	− 0.006	0.004	1.89E-01	− 0.028	0.007	5.93E-05	6.09E-03	*	*DMD*^‡^	Xp21.1
	rs138641025	A/G	0.02	3	− 0.020	0.006	9.89E-04	− 0.010	0.007	1.75E-01	− 0.043	0.011	4.74E-05	9.34E-03	*	*TMEM47*	Xp21.1
	rs190159509	T/C	0.05	3	− 0.002	0.005	6.50E-01	0.010	0.006	7.82E-02	− 0.040	0.010	5.07E-05	1.13E-05	*	*ITM2A*	Xq21.1
	rs142755000	A/G	0.03	3	− 0.028	0.006	3.36E-06	− 0.028	0.007	1.68E-04	− 0.028	0.011	7.07E-03	9.81E-01		*HMGN5*	Xq21.1
	rs185387095	T/A	0.01	2	− 0.039	0.009	2.82E-05	− 0.060	0.012	2.15E-06	− 0.029	0.015	5.51E-02	1.15E-01		*HMGN5*^‡^	Xq21.1
	**rs145089551**	**A/G**	**0.01**	**2**	− 0.033	0.009	1.50E-04	− **0.060**	**0.012**	**4.95E-07**	− 0.013	0.014	3.45E-01	9.55E-03	*	***SH3BGRL***	**Xq21.1**
	rs73230918	A/G	0.05	3	− 0.010	0.004	1.90E-02	− 0.002	0.005	6.21E-01	− 0.031	0.008	8.53E-05	1.82E-03	*	*POU3F4*	Xq21.1
	**rs5988060**	**A/G**	**0.36**	**3**	− 0.005	0.002	1.37E-02	− 0.001	0.002	8.02E-01	− **0.016**	**0.004**	**1.65E-05**	3.89E-04	*	***HTR2C***	**Xq23**
	rs185903771	A/G	0.04	3	− 0.015	0.005	1.22E-03	− 0.025	0.005	5.29E-06	0.010	0.008	2.28E-01	4.36E-04	*	*ARHGAP36*	Xq26.1
Emphysema	**rs147204746**	**G/A**	**0.01**	**2**	− **0.511**	**0.120**	**2.36E-05**	− 0.506	0.124	4.93E-05	− 0.565	0.318	7.48E-02	8.63E-01		***FRMPD4***^‡^	**Xp22.2**
	**rs184018661**	**G/A**	**0.02**	**3**	− 0.130	0.057	2.37E-02	0.030	0.060	6.27E-01	− **0.485**	**0.114**	**2.45E-05**	6.53E-05	*	***TAB3***	**Xp21.2**
	rs5905883	T/A	0.33	3	0.072	0.018	9.57E-05	0.075	0.020	1.41E-04	0.061	0.040	1.26E-01	7.55E-01		*DUSP21*	Xp11.3
	rs12845098	G/T	0.17	3	0.022	0.024	3.74E-01	− 0.043	0.026	9.91E-02	0.207	0.052	9.28E-05	2.15E-05	*	*TBX22*	Xq21.1
	rs184737280	T/C	0.02	3	− 0.216	0.056	1.50E-04	− 0.102	0.062	1.00E-01	− 0.461	0.114	6.20E-05	5.66E-03	*	*TCEAL1*	Xq22.2
	rs187046350	A/G	0.02	3	− 0.090	0.083	2.82E-01	0.125	0.088	1.58E-01	− 0.768	0.183	3.38E-05	1.23E-05	*	*GUCY2F*	Xq22.3
	**rs2748634**	**A/G**	**0.43**	**3**	0.052	0.016	1.12E-03	**0.080**	**0.017**	**2.61E-06**	− 0.028	0.033	4.07E-01	3.66E-03	*	***SLITRK2***	**Xq27.3**
	rs149254153	G/T	0.02	3	− 0.124	0.067	6.15E-02	0.048	0.068	4.93E-01	− 0.612	0.147	3.61E-05	4.78E-05	*	*FMR1-AS1*	Xq27.3

Summary of the top suggested associations in the XWAS meta-analysis in COPD as well as related phenotypes of spirometric lung function and emphysema measured by quantitative chest CT. Included are the top variants per annotated gene in at least one population strata for a given phenotype with P value ≤ 10^–6^ if present, otherwise ≤ 10^–5^ or less (Note: ≤ 10^–5^ was used for XWAS of all subjects emphysema, females FEV_1_, FEV_1_/FVC, and emphysema). Bold rows are top suggested association for that phenotype and population strata. ^*^ P < 0.05 for significant sex-difference. ^‡^ The variant is in the gene and intronic. ^§^ The variant is in the gene and a coding variant. Abbreviations: *XWAS* X-chromosome association study; COPD chronic obstructive pulmonary disease; *SNP* single nucleotide polymorphism; *EA* effect allele; *EAF* effect allele frequency; *OR* odds ratio; *CI* confidence interval; *FEV1* Forced expiratory volume in one second; *L* liters; *FVC* forced vital capacity

### Sex differences

Testing for sex difference compared effect estimates among the top suggested associations in sex-stratified meta-analysis for males and females (Table 2). Testing was run in 32 variants, which included 28 unique variants annotated to 25 unique genes. There were significant sex differences identified among 20 of the variants, which implicated 17 unique genes based on annotation to the closest gene. These variants were found across all four phenotypes, including 7 variants with larger effect in males and 13 variants with larger effect in females. There were two genes, *DMD* and *POU3F4*, both implicated by more than one variant among the top suggested associations, where the sex-effect was different dependent on the variant.

### ACE2

Recent attention has come to the X chromosome gene *ACE2* and its role in SARS-CoV-2 susceptibility. SARS-CoV-2 severity has been shown to vary by sex, and there is increased risk for severe disease in those with chronic respiratory conditions including COPD [29, 30]. Meta-analysis results for variants in *ACE2*, located at Xp22.2 (base pair region 15579156–15620192), were examined and top associations with each phenotype and sex strata are in Additional file 3: Table S2. None of the *ACE2* variants approached genome-wide significance.

### Replication

Examination of ten X chromosome variants previously discovered to have genome-wide significant associations with lung function in prior studies included one variant, rs142755000, that was among the top suggested associations in this meta-analysis XWAS for FEV_1_ ($\beta$ − 0.134, *SE* 0.029, *p* 4.30 × 10^–06^) and FEV_1_/FVC ($\beta$ − 0.028, *SE* 0.006, *p* 3.36 × 10^–06^), with the effect allele A having a frequency of 0.03 (Additional file 4: Table S3). This was comparable to results of Zhao et al. who found, among 5768 subjects in a COPD-enriched population of White race including COPDGene, that rs142755000 was a genome-wide association for FEV_1_ ($\beta$ − 0.18, *SE* 0.03, *p* 3.58 × 10^–08^), also having effect allele of A with a frequency of 0.03, and a similar appearance to the locus zoom plot [17]. Description of annotation for rs142755000 is in the supplement (Additional file 1).

## Discussion

This meta-analysis is the first comprehensive examination of markers on the X chromosome specifically testing for association with COPD and COPD-related phenotypes of pulmonary function and emphysema in three case–control populations. Using stringent data quality control and statistical methods specific to the X chromosome, we identified a genome-wide significant association with FEV_1_/FVC ratio for one variant near *TMSB4X*. There were additional suggestive associations with COPD-related phenotypes in analyses of all subjects and in sex-stratified XWAS. These findings emphasize the importance of properly including X chromosome variants in association analyses. They also support the important role sex differences play in the pathobiology of complex lung disease, including factors such as escape from XCI as seen with *TMSB4X*, and sex differences in effect size, as seen among 20 of the top suggested variants in stratified analysis.

Genetic risk factors for COPD have only been partially determined [2]. The X chromosome may contribute additional genetic risk that could explain some of the missing heritability for this complex disease not accounted for by autosomal GWAS results [3, 8]. COPD is a heterogeneous disease that also shows sex specific pathobiology [10]. Females have been suggested to have earlier onset of disease, and greater lung function decline per daily number of cigarettes smoked, while males may have more emphysema by quantitative imaging [9, 11]. An autosomal variant previously studied by Hardin et al. found sex-specific associations with COPD [4]. A larger GWAS by Sakornsakolpat et al. where 82 autosomal COPD variants were identified did not show sex-specific effects; this question may be better answered by examining the X chromosome [3].

The X chromosome has been included in prior lung function GWAS, although not routinely, and in that setting has been included in COPD investigations, though there has not previously been a direct XWAS of COPD and related phenotypes [14–17]. A lung function GWAS including the X chromosome by Soler Artigas et al. identified one variant at genome-wide significance that does not replicate in this study [14]. Wyss and colleagues included the X chromosome in their assessment of lung function and did not find any significant associations [15]. A large GWAS by Shrine et al. looked at a genetic risk score for lung function, including examination of the X chromosome with identification of five variants at genome-wide significance in the UK Biobank; however, these variants did not replicate in SpiroMeta and no variants met the study threshold for inclusion in their genetic risk score [16]. None of these variants replicated in our current XWAS. Most recently, Zhao and colleagues examined pulmonary function and COPD in whole genome sequencing data, identifying four variant signals on the X chromosome for FVC and one for FEV_1_, rs142755000, which was in a COPD-enriched stratum [17]. This FEV_1_ variant near *HMGN5* was among the top suggested associations in our current meta-analysis XWAS for FEV_1_ and FEV_1_/FVC.

In this analysis we demonstrated the importance of proper inclusion of the X chromosome as it harbors variants with association and suggestive association with COPD and COPD-related phenotypes. A variant near *TMSB4X* reached the genome-wide significance threshold for association with lung function in all subjects for FEV_1_/FVC and showed a suggestive association with FEV_1_. In the emphysema XWAS, the top suggestive association in all subjects was a variant in *FRMPD4*, located 332 kb upstream of the *TMSB4X* variant in the Xp22.2 locus; both *FRMPD4* and *TMSB4X* escape XCI [31]. *TMSB4X* encodes an actin sequestering protein, thymosin $\beta$ 4, that plays a role in regulation of actin polymerization and is important in organization of the cytoskeleton, as well as being involved in cell proliferation, migration, and differentiation [32]. *TMSB4X* is expressed equivalently in both male and female lung tissue but has been observed to have male-biased expression in skin, adipose, and kidney [33–35]. A comprehensive analysis of transcriptome sequencing data in COPD lung tissue demonstrated decreased expression in COPD compared to normal tissue based on both RNA-seq and quantitative real-time PCR [36]. Thymosin $\beta$ 4 and one of its methionine oxidation products, thymosin $\beta$ 4 sulfoxide, has been found at increased levels in bronchioalveolar lavage fluid of smokers [37]. In smokers, methionine oxidation plays a role in $\alpha$(1)-antitrypsin inactivation and pathologic lung remodeling [37, 38]. Thymosin $\beta$ 4 is thought to limit inflammation through autophagy and has been found to have a protective effect in interstitial lung diseases including scleroderma, bleomycin-induced lung damage, and reperfusion-induced acute lung injury [37, 39–41]. A variant near *TMSB4X* has been associated with risk of childhood onset asthma in the UK Biobank; though it is not found in linkage disequilibrium with the variant identified in this study the direction of effect is the same [42, 43].

There were additional suggestive associations found among the all-subject XWAS. In this lung function XWAS, variants from the Xq21.1 locus including in *HMGN5* and near *SH3BGRL* were implicated, which are both expressed in lung tissue [33]. A variant in *HMGN5*, rs185387095, was a top suggested association in this study, and a nearby variant in rs142755000 in linkage disequilibrium was also suggested; rs142755000 was identified in association with FEV_1_ in prior whole genome sequence analysis of COPD-enriched White race populations including COPDGene [17]. *HMGN5* modulates cellular transcription and, in a murine model, mutations in *HMGN5* are associated with a lung function phenotype on pulmonary function tests as well as an emphysema-like phenotype [44].

The sex-stratified analysis of COPD-related phenotypes provides additional revealing information and suggests functional associations based on size and direction of effect not identified in XWAS of all subjects. There was a larger effect in females found in sex-stratified testing among top suggested variants: two for COPD, one for FEV_1_, five for FEV_1_/FVC and five for emphysema. This included different directions of effect among one variant for lung function near *ITM2A* and four variants for emphysema near *TAB3, TBX22*, *GUCY2F*, and *FMR1-AS1*. There was a larger effect in males found in sex-stratified testing among top suggested variants: one for COPD, five for lung function, and one for emphysema. This included different directions of effect among two variants for lung function, one in *OPHN1* and one near *ARHGAP36,* as well as one variant for emphysema near *SLITRK2*. Further discussion of suggestive associations can be found in the Additional file 1).

One source of potential disease pathology for some of these variants is escape from XCI [45, 46]. Mammalian females carry two copies of chromosome X, and the majority of X-linked human genes are subject to XCI, where one of the two copies is silenced. However, at least 15–23% of genes escape XCI to some extent, and thus both X chromosome genes are expressed, with a variable continuum of expression likely due to epigenetic effects [31, 34, 47]. XCI is not random, with the majority of escape genes found on the short arm of chromosome X, a region enriched for male-biased and female-biased genes [34, 35, 48]. This is likely related to the evolutionary history of the sex chromosomes where the short arm of the X chromosome is a recent addition to an ancestral chromosome [48]. The impact of XCI is complex and variable across individuals and tissue types, and without functional studies it is not possible to know if the closest annotated genes are causal [34, 35]. *TMSB4X*, the closest gene to the top variant implicated in this analysis, is known to escape XCI, though there is not a significant sex difference in the current analysis. Evidence of escape from XCI has been reported for other genes implicated by suggested associations in or near *DMD, HTR2C,* and *GUCY2F*, which demonstrate a significant sex difference in our analysis, as well as *SH3KBP1* and *FRMPD4*, which do not have significant sex differences in the current analysis [31, 32, 34, 35, 49, 50]. Future investigation of gene expression and methylation patterns together with genetic variation may reveal pathologic relevance for these genes that escape XCI despite nominal evidence for sex-specific genetic association.

Escape from XCI results in sex biases in gene expression and has implications for the role of the X chromosome in human diseases, including intellectual disability, autoimmune disease, and cancer [34, 35, 45, 46, 48, 51]. It has been suggested that escape from XCI is a mechanism for respiratory disease pathology in COPD and that escape from XCI influences lung tissue transcription [52]. *POU3F4* was the only suggested variant in the current analysis that is a transcription factor. In our published sex-specific network analysis of gene expression data in normal lung tissue, *POU3F4* demonstrated sexed biased targeting of 15 X chromosome genes, 14 of which escape XCI, including two important XCI regulators, *XIST* and *JPX* (Additional file 1: Figure S5) [53, 54]. This points to sex-biased genetic variants as one mechanism implicated in sex-biased transcriptional targeting.

We implemented X chromosome-specific genetic association testing for our analyses, using parameters conforming to Clayton’s method for analysis to account for XCI in females and male X chromosome hemizygosity [6, 27]. Hickey and colleagues compared Clayton’s method to autosomal methods and alternate X chromosome methods and found this approach provided the best power to detect an association [7, 27]. This method has been used in several GWAS that identified significant association between X chromosome markers and complex disease traits [14, 55, 56].

Prior COPD GWAS have excluded genomic information from the X chromosome in their analyses [3, 16, 24]. Although individual reasons for excluding these data are not known, many studies exclude X chromosome genetic variants because of the statistical difficulties inherent to testing for association with the sex chromosomes. We implemented X-specific approaches to both quality control and statistical analysis to improve both accuracy and power to detect an association. Larger studies of X chromosome markers will be required to reliably break the threshold of genome-wide significance on the X for COPD and related phenotypes, but we feel that our results represent an important step in determining associations involved in smoking-related obstructive lung disease.

Our study has several limitations. Since the study cohorts are limited to smokers, we were not able to assess risk variants or effect modification among non-smokers and our results may not generalize to non-smokers. Clayton’s method provides a statistical approach to random XCI, but some areas of the X undergo non-random inactivation [27]. Future studies including allele-specific methylation will be helpful to directly investigate the effect of XCI more thoroughly. Variants with minor allele frequency of 1% or greater were included in this analysis, though the allele frequency of some of the suggestive associations was near 1%, which increases the likelihood that these suggestive associations are driven by a small number of subjects; low frequency variants should be interpreted with caution. We were unable to replicate all the previously discovered genome-wide significant associations with lung function, which may be related to differences in phenotypes examined (five variants were for FVC only, which was not examined in the current study), study design, power, and patient populations including the inclusion of current and former smokers only [14, 16, 17]. The top hits described in this study were not replicated in another population, which would be an interesting future direction. Despite these limitations, we did identify several intriguing biologically plausible genes that could play a potential role in COPD; additional investigation of allosomal genetic variation and lung disease is imperative.

## Conclusions

In summary, these results represent a comprehensive analysis of markers on the X chromosome for association with COPD and related phenotypes including emphysema in three COPD case–control cohorts. We identified one genome-wide significant variant and several promising associations between markers on the X chromosome that may contribute to sex differences in COPD. Among 33 top suggested variants there were 20 unique variants that had significant sex differences in stratified analysis, seven with larger effect in males and thirteen with larger effect in females. Among the 25 top genes implicated with suggestive associations in this study, at least six have evidence for escape from XCI, including the top genome-wide association near *TMSB4X*. XCI may be an important contributor to disease pathology, though the impact of XCI is complex and variable. Detecting variants associated with complex traits is inherently difficult and requires large sample sizes for males and females to address the statistical complexities of studying the X chromosome. Genetic association studies in human lung disease should routinely consider systematic interrogation of X chromosome variants as these may reveal new genes for sex-specific diagnostic and therapeutic approaches to COPD.

## Supplementary Information

**Additional file 1: Text and Figures S1–S5. **Supplementary text for the methods (study phenotype, quality control, imputation, significance and suggestive thresholds, annotation), results (power to detect associations, annotation of rs142755000), and discussion (suggestive associations) as well as related references. **Figure S1.** a: COPDGene XWAS in Stratified Populations All Subjects. b: COPDGene XWAS in Stratified Populations Males. c: COPDGene XWAS in Stratified Populations Females. **Figure S2.** Meta-analysis XWAS in Stratified Population. **Figure S3.** Meta-analysis Locus Plots of Top Suggested Associations in COPD Related Phenotypes. **Figure S4.** Meta-analysis Quantile–Quantile and Manhattan Plots. **Figure S5**. Sex differential edge weights connecting the transcription factor *POU3F4* in GTEx Lung Tissue.

**Additional file 2: Table S1. **XWAS Meta-analysis All Top Suggested Associations At 10^-6^ Or Less If Present, Otherwise Top Suggested Associations At 10^–5^ Or Less

**Additional file 3: Table S2. **XWAS Meta-analysis Top *ACE2* Xp22.2 Variants

**Additional file 4: Table S3.** Replication Examination Of Associations From Other Studies In This XWAS Meta-analysis

## Data Availability

The datasets generated and/or analyzed during the current study are are publicly available in dbGaP for COPDGene (accession number phs000179.v6.p2, https://www.ncbi.nlm.nih.gov/projects/gap/cgi-bin/study.cgi?study_id=phs000179.v6.p2) and ECLIPSE (accession number phs001252.v1.p1, https://www.ncbi.nlm.nih.gov/projects/gap/cgi-bin/study.cgi?study_id=phs001252.v1.p1).

## Abbreviations

β: Beta
CI: Confidence interval
COPD: Chronic obstructive pulmonary disease
COPDGene: The Genetic Epidemiology of COPD Study
CT: Computed tomography
EA: Effect allele
EAF: Effect allele frequency
ECLIPSE: Evaluation of COPD Longitudinally to Identify Predictive Surrogate Endpoints Study
Emphysema: Quantitative emphysema
FEV_1_: Forced expiratory volume in one second
FVC: Forced vital capacity
GenKOLS: Genetics of COPD in Norway Study
GOLD: Global Initiative for Chronic Obstructive Lung Disease
GWAS: Genome-wide association studies
L: Liters
OR: Odds ratio
QC: Quality control
SD: Standard deviation
SE: Standard error
SNP: Single nucleotide polymorphism
XCI: X chromosome inactivation
XWAS: X-chromosome-wide association study
