# Supplementary material for: X chromosome associations with chronic obstructive pulmonary disease and related phenotypes: an X chromosome-wide association study
Source: Respir Res. 2023 Feb 1;24:38. doi: 10.1186/s12931-023-02337-1 (PMC9891756; doi:10.1186/s12931-023-02337-1)
Supplement: Supplementary file 1 — Additional file 1: Text and Figures S1–S5. Supplementary text for the methods (study phenotype, quality control, imputation, significance and suggestive thresholds, annotation), results (power to detect associations, annotation of rs142755000), and discussion (suggestive associations) as well as related references. Figure S1. a: COPDGene XWAS in Stratified Populations All Subjects. b: COPDGene XWAS in Stratified Populations Males. c: COPDGene XWAS in Stratified Populations Females. Figure S2. Meta-analysis XWAS in Stratified Population. Figure S3. Meta-analysis Locus Plots of Top Suggested Associations in COPD Related Phenotypes. Figure S4. Meta-analysis Quantile–Quantile and Manhattan Plots. Figure S5. Sex differential edge weights connecting the transcription factor POU3F4 in GTEx Lung Tissue. [file 12931_2023_2337_MOESM1_ESM.pdf]

SUPPLEMENT

X Chromosome Genetic Associations with Chronic Obstructive Pulmonary Disease and Related Phenotypes

Lystra P Hayden, Brian D Hobbs, Robert Busch, Michael H Cho, Ming Liu, Camila M Lopes-Ramos, David A Lomas, Per Bakke, Amund Gulsvik, Edwin K Silverman, James D Crapo, Terri H Beaty, Nan M Laird, Christoph Lange, Dawn L DeMeo.

METHODS

Study phenotype

Subjects were current and former smokers with at least a 10 pack-year smoking history in COPDGene and ECLIPSE, and at least a 2.5 pack-year smoking history in GenKOLS. Subjects completed a modified American Thoracic Society respiratory questionnaire and standardized spirometry, as well as chest CT, and provided blood for DNA extraction and genetic analysis (1, 2).

Quality control

Study based subject-level data cleaning removed subjects with sex discrepancies including chromosomal aneuploidies, and tested for excess or deficiency of heterozygosity (inbreeding coefficients, F-test absolute value > 0.2) (3). Study based variant-level cleaning was conducted separately in male and female datasets. Variants were excluded if they had minor allele frequency < 1%, variant level missingness > 2%, differential missingness between sexes of ≥ 2%, or for a significant departure from Hardy-Weinberg equilibrium in females ($p < 1 \times 10^{-4}$). The data cleaned by sex was merged, and only variants passing quality control in both sexes for that study were retained for XWAS.

Imputation

Data from subjects and variants passing quality control were used for imputation of untyped variants based on the Haplotype Reference Consortium reference panel (version r1.1 2016) via the Michigan Imputation Server v1.0.2 (https://imputationserver.readthedocs.io/en/latest/pipeline/) (4). Imputed posterior genotype dosage probabilities were filtered for imputation accuracy (INFO-score) and removed for $R^2 < 0.5$ (3).

Significance and suggestive thresholds

The primary threshold for identifying genome-wide significant associations was a p-value for association $p < 5 \times 10^{-8}$ (5, 6). We examined additional suggestive associations, defined as p-values at the level of 10^{-6} or less if associations were present at that level, and at the level to 10^{-5} if no other associations were observed.

Annotation
Annotation of variants was performed by assessing the closest gene to each variant by distance using National Center for Biotechnology Information databases dbSNP/Gene, UCSC Genome Browser, Ensembl, LDlink, and LocusZoom (7-11). Distance based annotation does not imply function and other genes in the region should be considered for functional investigations.

RESULTS

Power to detect associations
The genotype relative risk for rare events, including a single nucleotide polymorphisms (SNP), is approximately the odds-ratio (OR). In this study, assuming a COPD prevalence of 10%, while using a genome-wide significance level of p 5 x 10^{-8}, and a disease allele frequency threshold equal to the effect allele frequency (EAF) of < 1%, the current meta-analysis with 5382 cases and 3501 controls (case to control ratio 1.537) has 90% power to detect a variant with an OR of 2.095, 10% power to detect an OR of 1.639, and < 0.1% power to detect an OR in the range of 1.000 - 1.320 (12, 13).

In Table 2 we present the top suggested COPD association from the meta-analysis, including top variants found in at least one population strata. The EAF range is 0.01 – 0.39, OR range is 0.65 – 3.83, and Pvalue range is 0.897 – 2.65 x 10^{-6}. For the top COPD variant among all subjects, in rs138704174 with EAF 3%, there is 85.4% power to detect this association with OR of 1.58 and p 5 x 10^{-8}. For the lowest EAF variant for COPD among all subjects, rs150086151 with EAF 1%, there is 3.5% power to detect this association with OR of 1.55 at p 5 x 10^{-8}. For a theoretical variant in this COPD meta-analysis with EAF 1% there is < 1% power to detect an association with an OR < 1.316 at p 5 x 10^{-8}.

To improve power with the same COPD prevalence (10%) and significance level ($p < 5 \times 10^{-8}$), an idealized study would have a case to control ratio of 1.00 and would require 100,000 cases and 100,000 controls to have 90% power to detect a variant with EAF of 1% and an OR of 1.203.

Annotation of rs142755000
The sex-stratified analysis by Zhao et al. found that rs142755000 reached genome-wide significance for FEV_1. Zhao found it has the same direction of effect in males and females, but a notably larger effect in males, which was not seen in our current study. We annotated rs142755000 to *HMGN5* and a nearby top suggested variant we identified in *HMGN5*, rs185387095, did have significant sex-differences in this XWAS for FEV_1 with larger effect in males (β males -0.039, β females -0.029, sex-difference p 3.30 x 10^{-02}). The closest gene to rs142755000 is *BRWD3*, 149kb upstream. In this study we annotated rs142755000 to *HMGN5*, which is 155kb downstream (Table 2). This annotation to *HMGN5* was made due to the fact that another top suggested association in this study, rs185387095, is found in *HMGN5*, and rs142755000 is in linkage disequilibrium (LD) at R^2 0.52 (11). Additionally, there are other LD variants within the same recombination hotspot that includes *HMGN5* and are in and near rs142755000 (supplement Figure 3).

DISCUSSION

Suggestive associations

In COPD there was a suggestive association in *SH3KBP1*, a gene that escapes XCI, encoding a protein that facilitates protein-protein interactions and has been implicated in cellular processes including cell adhesion, cytoskeletal rearrangement, apoptosis, endocytosis, and it has been shown to play a role in maturation of alveolar epithelial cells and surfactant production in mice (7, 14-16). *SH3KBP1* is expressed in lung tissue and exhibits sex-biased gene expression in whole blood (17, 18).

In lung function XWAS, variants in Xp11.21 were implicated including the dense region in *FOXR2/*near *RRAGB*/near *PAGE5. FOXR2* is a member of the FOX superfamily of genes known to interact with β-catenin and to play a role in epithelial-mesenchymal transition of cancer cells, and in non-small cell lung cancer (NSCLC). *FOXR2* expression inactivates the Wnt/β-catenin pathway (19, 20). *RRAGB*, a gene expressed in lung tissue, is part of a large family of signal transducers, and high expression of *RRAGB* has been found to predict good survival in NSCLC (7, 18, 21). *PAGE5* is part of family of proteins expressed in some fetal tissues as well as in a variety of tumors, and it encodes a protein that may protect cells from programed cell death (22).

Among top suggestive associations in females were a number of genes that are expressed in lung tissue and have interesting implications in disease pathobiology (18). *LINC0259* is a long noncoding RNA that mediates TGF-β signaling and has been implicated in lung cancer cell migration and invasion (18, 23). *TMEM47* encodes a highly conserved protein that is a member of the PMP22/EMP/claudin family important in cell morphology that is involved in localization of tight junction proteins and actomyosin structure (7, 24, 25). *ITM2A* has been implicated in ankylosing spondylitis where it is differentially expressed by CD4+ T cells and is involved in T cell activation (26, 27). *TAB3* encodes a protein that functions in the NF-κB signaling pathway, which plays a role in response to pro-inflammatory cytokines TNF or IL-1. It has been reported to be involved in signaling events in pathogenesis or progression of idiopathic pulmonary fibrosis, where it is thought to alter immune response, tissue repair, and fibrosis (28).

DMD, a top suggestive association for COPD for males as well as for FEV_1/FVC for females, encodes dystrophin and is the largest gene identified in humans; it has been associated with an extensive number of traits (7, 15, 16, 29). *DMD* is known to escape X chromosome inactivation, demonstrate sex-biased gene expression in lung tissue with a female bias, and have female-biased expression in numerous other tissues (15, 17, 30). Dystrophin is present in cardiac and cytoskeletal muscle and mutations in *DMD* lead to Duchenne's and Becker's muscular dystrophies, X-linked recessive disorders that manifests only in males and results from decreased dystrophin production (31). Duchenne's muscular dystrophy patients have early mortality related to respiratory muscle weakness, with decreased muscle strength leading to hypoventilation.

REFERENCES

1. COPDGene. COPDGene, Phase 1 Study Documents [Available from: http://www.copdgene.org/phase-1-study-documents.
2. Ferris BG. Epidemiology Standardization Project (American Thoracic Society). Am Rev Respir Dis. 1978;118(6 Pt 2):1-120.
3. Konig IR, Loley C, Erdmann J, Ziegler A. How to include chromosome X in your genome-wide association study. Genetic epidemiology. 2014;38(2):97-103.
4. McCarthy S, Das S, Kretzschmar W, Delaneau O, Wood AR, Teumer A, et al. A reference panel of 64,976 haplotypes for genotype imputation. Nature genetics. 2016;48(10):1279-83.
5. Hoggart CJ, Clark TG, De Iorio M, Whittaker JC, Balding DJ. Genome-wide significance for dense SNP and resequencing data. Genetic epidemiology. 2008;32(2):179-85.
6. Chu X, Shen M, Xie F, Miao XJ, Shou WH, Liu L, et al. An X chromosome-wide association analysis identifies variants in GPR174 as a risk factor for Graves' disease. J Med Genet. 2013;50(7):479-85.
7. NCBI Resource Coordinators. Database resources of the National Center for Biotechnology Information. Nucleic acids research. 2016;44(D1):D7-19.
8. Pruim RJ, Welch RP, Sanna S, Teslovich TM, Chines PS, Gliedt TP, et al. LocusZoom: regional visualization of genome-wide association scan results. Bioinformatics. 2010;26(18):2336-7.
9. Kent WJ, Sugnet CW, Furey TS, Roskin KM, Pringle TH, Zahler AM, et al. The human genome browser at UCSC. Genome Res. 2002;12(6):996-1006.
10. McLaren W, Gil L, Hunt SE, Riat HS, Ritchie GR, Thormann A, et al. The Ensembl Variant Effect Predictor. Genome Biol. 2016;17(1):122.
11. Machiela MJ, Chanock SJ. LDlink: a web-based application for exploring population-specific haplotype structure and linking correlated alleles of possible functional variants. Bioinformatics. 2015;31(21):3555-7.
12. Skol AD, Scott LJ, Abecasis GR, Boehnke M. Joint analysis is more efficient than replication-based analysis for two-stage genome-wide association studies. Nature genetics. 2006;38(2):209-13.
13. Buist AS, McBurnie MA, Vollmer WM, Gillespie S, Burney P, Mannino DM, et al. International variation in the prevalence of COPD (the BOLD Study): a population-based prevalence study. Lancet. 2007;370(9589):741-50.
14. Coulombe P, Paliouras GN, Clayton A, Hussainkhel A, Fuller M, Jovanovic V, et al. Endothelial Sash1 Is Required for Lung Maturation through Nitric Oxide Signaling. Cell Rep. 2019;27(6):1769-80 e4.
15. Zhang Y, Castillo-Morales A, Jiang M, Zhu Y, Hu L, Urrutia AO, et al. Genes that escape X-inactivation in humans have high intraspecific variability in expression, are associated with mental impairment but are not slow evolving. Mol Biol Evol. 2013;30(12):2588-601.
16. Cotton AM, Ge B, Light N, Adoue V, Pastinen T, Brown CJ. Analysis of expressed SNPs identifies variable extents of expression from the human inactive X chromosome. Genome Biol. 2013;14(11):R122.
17. Oliva M, Munoz-Aguirre M, Kim-Hellmuth S, Wucher V, Gewirtz ADH, Cotter DJ, et al. The impact of sex on gene expression across human tissues. Science. 2020;369(6509).
18. Consortium GT. The Genotype-Tissue Expression (GTEx) project. Nature genetics. 2013;45(6):580-5.

19. Zheng X, Lin J, Wu H, Mo Z, Lian Y, Wang P, et al. Forkhead box (FOX) G1 promotes hepatocellular carcinoma epithelial-Mesenchymal transition by activating Wnt signal through forming T-cell factor-4/Beta-catenin/FOXG1 complex. J Exp Clin Cancer Res. 2019;38(1):475.
20. Wang XH, Cui YX, Wang ZM, Liu J. Down-regulation of FOXR2 inhibits non-small cell lung cancer cell proliferation and invasion through the Wnt/beta-catenin signaling pathway. Biochem Biophys Res Commun. 2018;500(2):229-35.
21. Xie H, Xie C. A Six-Gene Signature Predicts Survival of Adenocarcinoma Type of Non-Small-Cell Lung Cancer Patients: A Comprehensive Study Based on Integrated Analysis and Weighted Gene Coexpression Network. Biomed Res Int. 2019;2019:4250613.
22. The Health Consequences of Smoking-50 Years of Progress: A Report of the Surgeon General. Reports of the Surgeon General. Atlanta (GA)2014.
23. Hao Y, Yang X, Zhang D, Luo J, Chen R. Long noncoding RNA LINC01186, regulated by TGF-beta/SMAD3, inhibits migration and invasion through Epithelial-Mesenchymal-Transition in lung cancer. Gene. 2017;608:1-12.
24. VanBelzen DJ, Malik AS, Henthorn PS, Kornegay JN, Stedman HH. Mechanism of Deletion Removing All Dystrophin Exons in a Canine Model for DMD Implicates Concerted Evolution of X Chromosome Pseudogenes. Mol Ther Methods Clin Dev. 2017;4:62-71.
25. Dong Y, Simske JS. Vertebrate Claudin/PMP22/EMP22/MP20 family protein TMEM47 regulates epithelial cell junction maturation and morphogenesis. Dev Dyn. 2016;245(6):653-66.
26. Lee YH, Song GG. Meta-analysis of differentially expressed genes in ankylosing spondylitis. Genet Mol Res. 2015;14(2):5161-70.
27. Tukiainen T, Pirinen M, Sarin AP, Ladenvall C, Kettunen J, Lehtimaki T, et al. Chromosome X-wide association study identifies Loci for fasting insulin and height and evidence for incomplete dosage compensation. PLoS Genet. 2014;10(2):e1004127.
28. Liu B, Jiang T, Hu X, Liu Z, Zhao L, Liu H, et al. Downregulation of microRNA30a in bronchoalveolar lavage fluid from idiopathic pulmonary fibrosis patients. Mol Med Rep. 2018;18(6):5799-806.
29. Mitchell JA, McCray AT. The Genetics Home Reference: a new NLM consumer health resource. AMIA Annu Symp Proc. 2003:936.
30. Tukiainen T, Villani AC, Yen A, Rivas MA, Marshall JL, Satija R, et al. Landscape of X chromosome inactivation across human tissues. Nature. 2017;550(7675):244-8.
31. Mosqueira M, Zeiger U, Forderer M, Brinkmeier H, Fink RH. Cardiac and Respiratory Dysfunction in Duchenne Muscular Dystrophy and the Role of Second Messengers. Med Res Rev. 2013.

Supplement Figure 1 a: COPDGene XWAS in Stratified Populations All Subjects

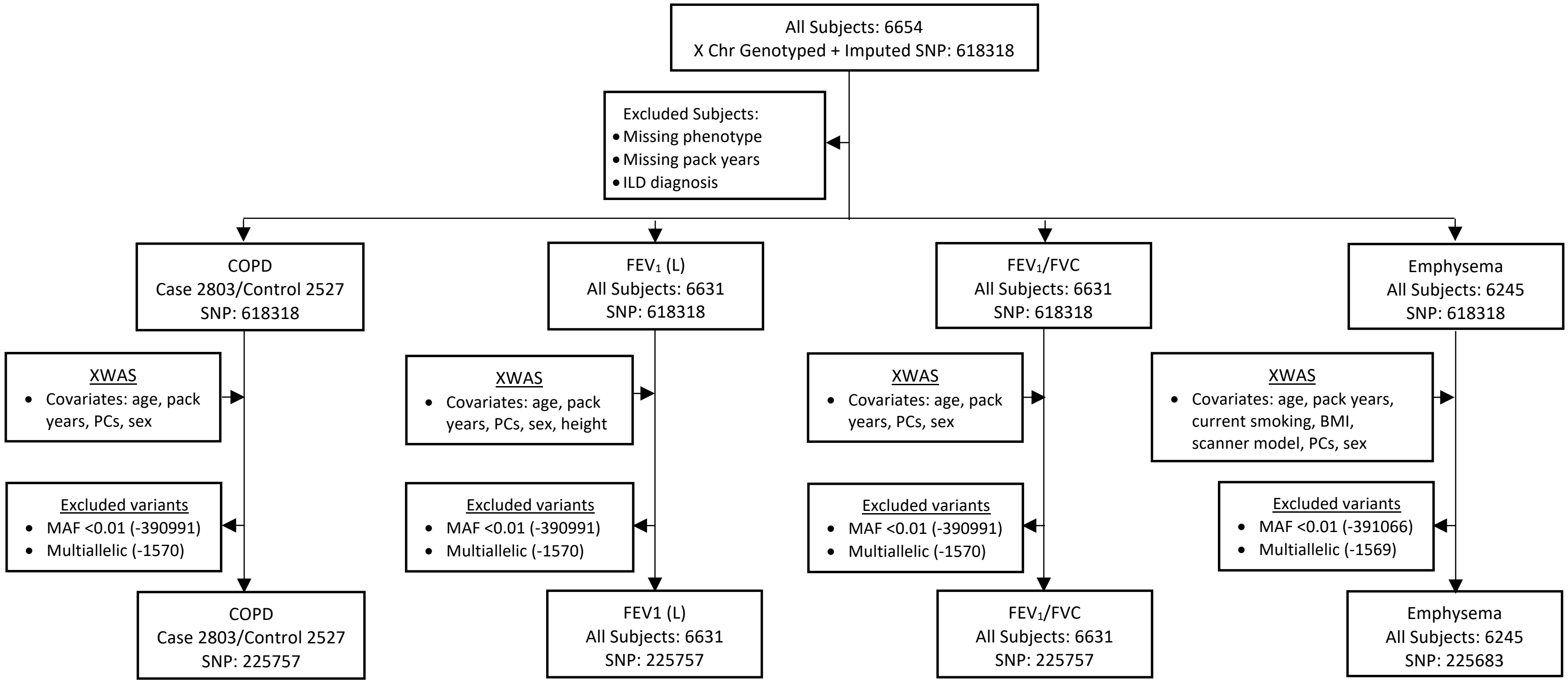

Supplement Figure 1 a Steps in XWAS for COPDGene all subjects. Numbers in parentheses represent variants removed. Abbreviations: *XWAS* X chromosome association study; *Chr* chromosome; *SNP* single nucleotide polymorphism; *ILD* interstitial lung disease; *COPD* chronic obstructive pulmonary disease; FEV_1 Forced expiratory volume in one second; *L* liters; *FVC* forced vital capacity; *PCs* principal components of genetic ancestry; *MAF* minor allele frequency.

Supplement Figure 1 b: COPDGene XWAS in Stratified Populations Males

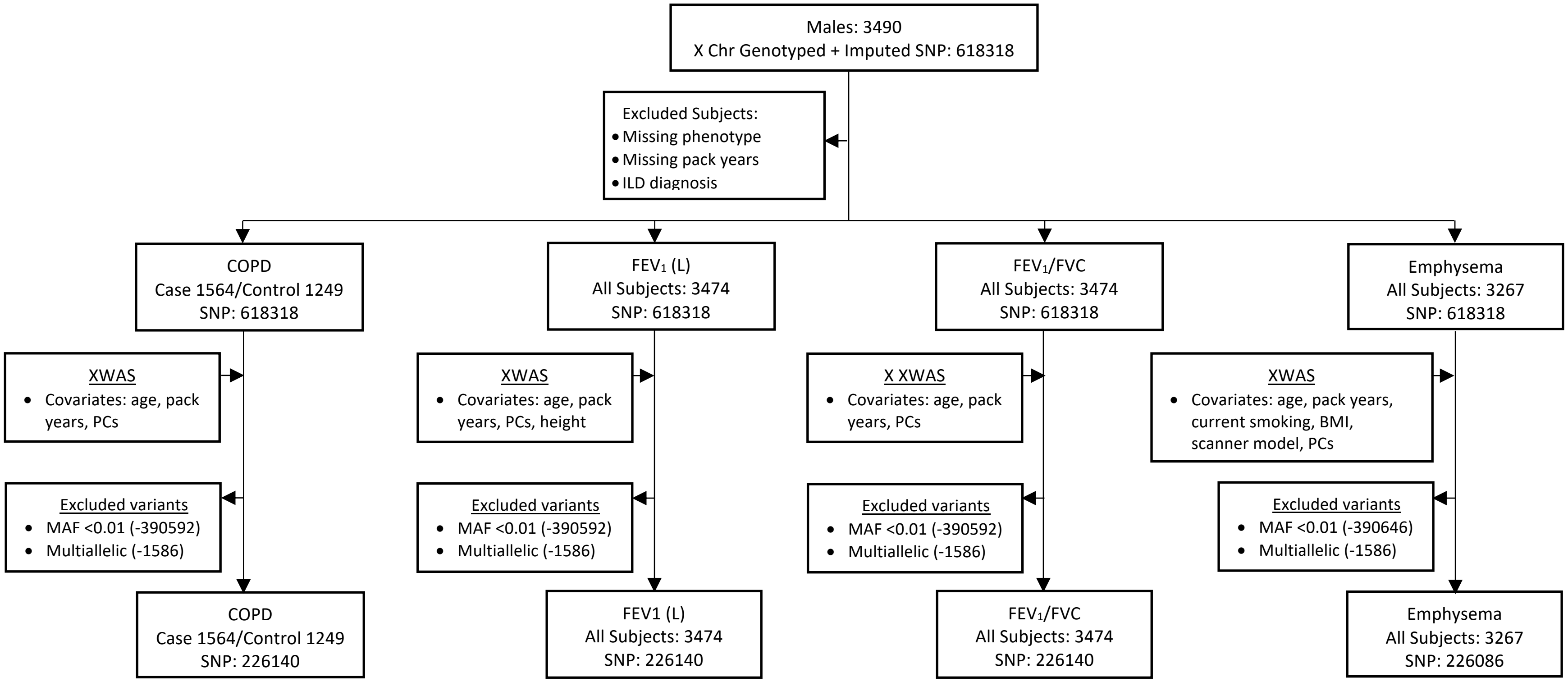

Supplement Figure 1 b Steps in XWAS for COPDGene male subjects. Numbers in parentheses represent variants removed. Abbreviations: *XWAS* X chromosome association study; *Chr* chromosome; *SNP* single nucleotide polymorphism; *ILD* interstitial lung disease; *COPD* chronic obstructive pulmonary disease; *FEV_1* Forced expiratory volume in one second; *L* liters; *FVC* forced vital capacity; *PCs* principal components of genetic ancestry; *MAF* minor allele frequency.

Supplement Figure 1 c: COPDGene XWAS in Stratified Populations Females

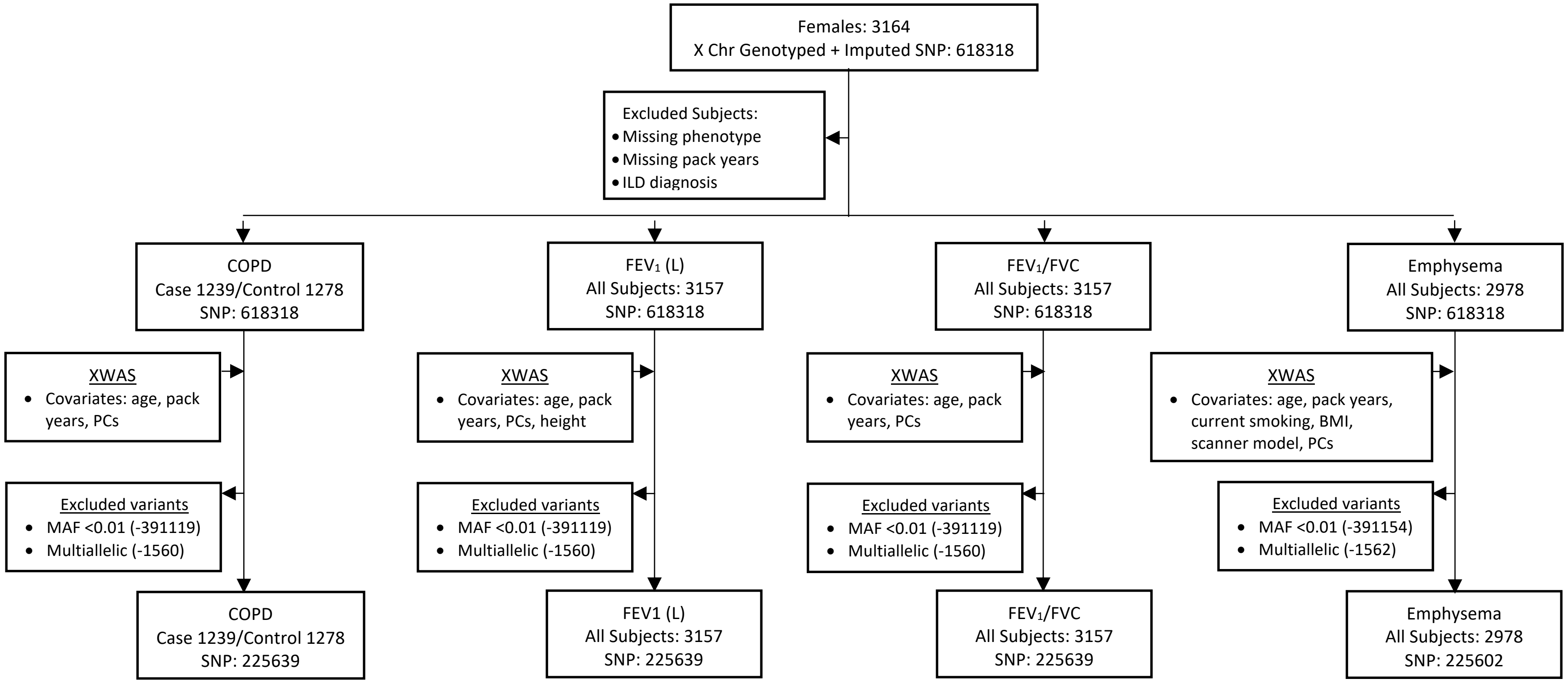

Supplement Figure 1 c Steps in XWAS for COPDGene female subjects. Numbers in parentheses represent variants removed. Abbreviations: *XWAS* X chromosome association study; *Chr* chromosome; *SNP* single nucleotide polymorphism; *ILD* interstitial lung disease; *COPD* chronic obstructive pulmonary disease; *FEV_1* Forced expiratory volume in one second; *L* liters; *FVC* forced vital capacity; *PCs* principal components of genetic ancestry; *MAF* minor allele frequency.

Supplement Figure 2: Meta-analysis XWAS in Stratified Population

ALL SUBJECTS

COPD

	Subjects: COPDGene	GenKOLS	ECLIPSE	TOTAL	Variants: COPDGene	GenKOLS	ECLIPSE
Case	2803	853	1726	**5382**	225757	225861	222412
Control	2527	805	169	**3501**			
	5330	1658	1895	**8883**			

↓ ← *plink meta-analysis*
238339
↓ → *Multiallelic (-2)*
238337
↓ → *Only in one study (-14383)*
223954

FEV1 (L)

	Subjects: COPDGene	GenKOLS	ECLIPSE	TOTAL	Variants: COPDGene	GenKOLS	ECLIPSE
	6631	1658	1904	**10193**	225757	225861	222499

↓ ← *plink meta-analysis*
238344
↓ → *Multiallelic (-2)*
238342
↓ → *Only in one study (-14346)*
223996

FEV1/FVC

	Subjects: COPDGene	GenKOLS	ECLIPSE	TOTAL	Variants: COPDGene	GenKOLS	ECLIPSE
	6631	1658	1904	**10193**	225757	225861	222498

↓ ← *plink meta-analysis*
238339
↓ → *Multiallelic (-2)*
238337
↓ → *Only in one study (-14338)*
223999

Emphysema

	Subjects: COPDGene	GenKOLS	ECLIPSE	TOTAL	Variants: COPDGene	GenKOLS	ECLIPSE
	6245	827	1502	**8574**	225683	226354	222574

↓ ← *plink meta-analysis*
239714
↓ → *Multiallelic (-4)*
239710
↓ → *Only in one study (-15764)*
223946

MALES

COPD

	Subjects: COPDGene	GenKOLS	ECLIPSE	TOTAL	Variants: COPDGene	GenKOLS	ECLIPSE
Case	1564	513	1155	**3232**	226140	226202	222038
Control	1249	404	96	**1749**			
	2813	917	1251	**4981**			

↓ ← *plink meta-analysis*
241757
↓ → *Multiallelic (-4)*
241753
↓ → *Only in one study (-17724)*
224029

FEV1 (L)

	Subjects: COPDGene	GenKOLS	ECLIPSE	TOTAL	Variants: COPDGene	GenKOLS	ECLIPSE
	3474	917	1258	**5649**	226140	226202	223607

↓ ← *plink meta-analysis*
241852
↓ → *Multiallelic (-4)*
241848
↓ → *Only in one study (-17580)*
224268

FEV1/FVC

	Subjects: COPDGene	GenKOLS	ECLIPSE	TOTAL	Variants: COPDGene	GenKOLS	ECLIPSE
	3474	917	1258	**5649**	226140	226202	223607

↓ ← *plink meta-analysis*
241852
↓ → *Multiallelic (-4)*
241848
↓ → *Only in one study (-17580)*
224268

Emphysema

	Subjects: COPDGene	GenKOLS	ECLIPSE	TOTAL	Variants: COPDGene	GenKOLS	ECLIPSE
	3267	482	975	**4724**	226086	226800	223680

↓ ← *plink meta-analysis*
244397
↓ → *Multiallelic (-2)*
244395
↓ → *Only in one study (-20288)*
224107

FEMALES

COPD

	Subjects: COPDGene	GenKOLS	ECLIPSE	TOTAL	Variants: COPDGene	GenKOLS	ECLIPSE
Case	1239	340	571	**2150**	225639	225766	221661
Control	1278	401	73	**1752**			
	2517	741	644	**3902**			

↓ ← *plink meta-analysis*
239431
↓ → *Multiallelic (-2)*
239429
↓ → *Only in one study (-16134)*
223295

FEV1 (L)

	Subjects: COPDGene	GenKOLS	ECLIPSE	TOTAL	Variants: COPDGene	GenKOLS	ECLIPSE
	3157	741	646	**4544**	225639	225766	222193

↓ ← *plink meta-analysis*
239502
↓ → *Multiallelic (-2)*
239500
↓ → *Only in one study (-16138)*
223362

FEV1/FVC

	Subjects: COPDGene	GenKOLS	ECLIPSE	TOTAL	Variants: COPDGene	GenKOLS	ECLIPSE
	3157	741	646	**4544**	225639	225766	222181

↓ ← *plink meta-analysis*
239493
↓ → *Multiallelic (-2)*
239491
↓ → *Only in one study (-16130)*
223361

Emphysema

	Subjects: COPDGene	GenKOLS	ECLIPSE	TOTAL	Variants: COPDGene	GenKOLS	ECLIPSE
	2978	345	527	**3850**	225602	226634	222026

↓ ← *plink meta-analysis*
241321
↓ → *Multiallelic (-2)*
241319
↓ → *Only in one study (-17935)*
223384

Supplement Figure 2 Steps in XWAS meta-analysis. Numbers in parentheses represent variants removed. Abbreviations: *XWAS* X chromosome association study; *COPD* chronic obstructive pulmonary disease; FEV_1 Forced expiratory volume in one second; *L* liters; *FVC* forced vital capacity.

Supplement Figure 3: Meta-analysis Locus Plots of Top Suggested Associations in COPD Related Phenotypes

COPD

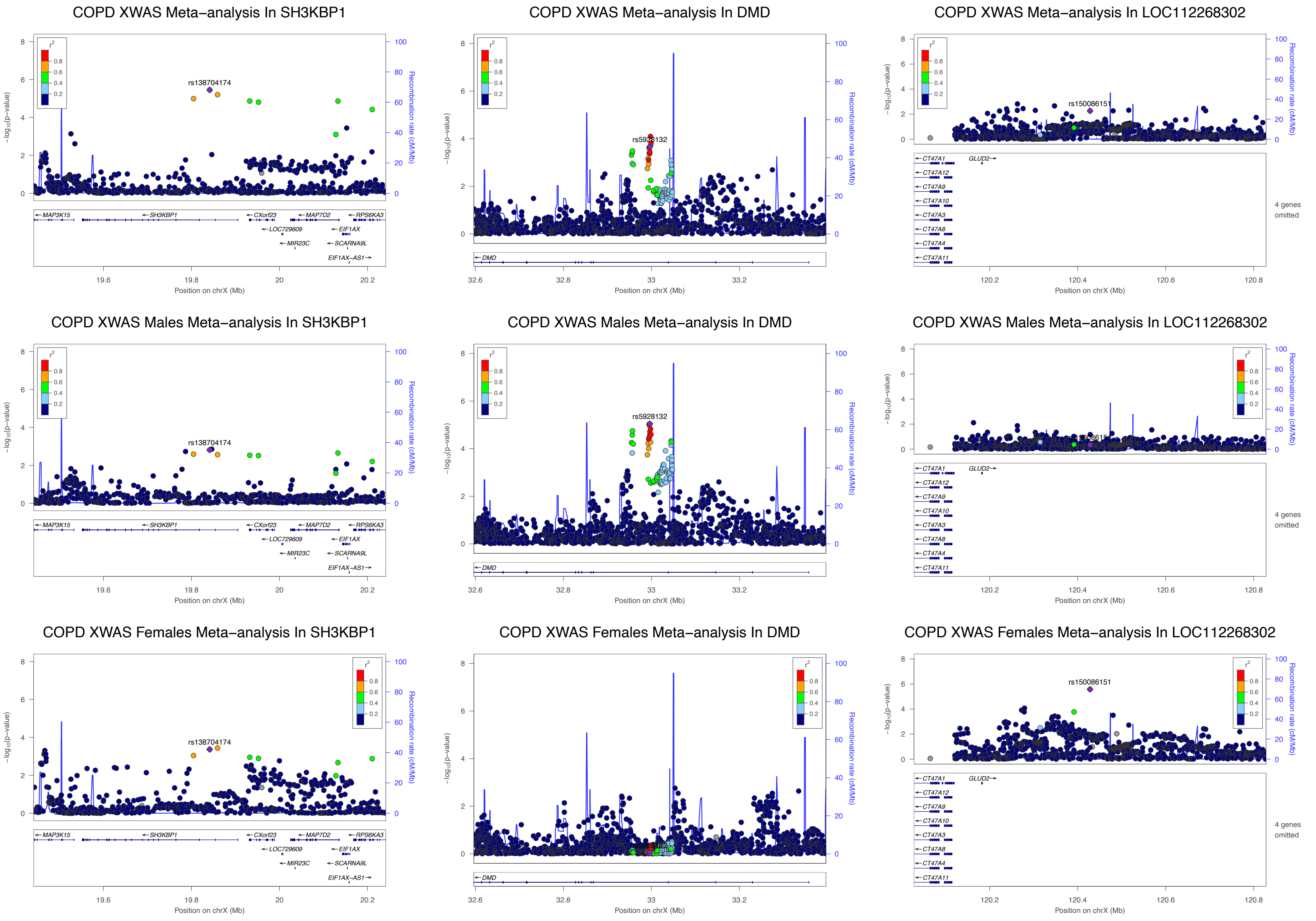

FEV$_1$, L

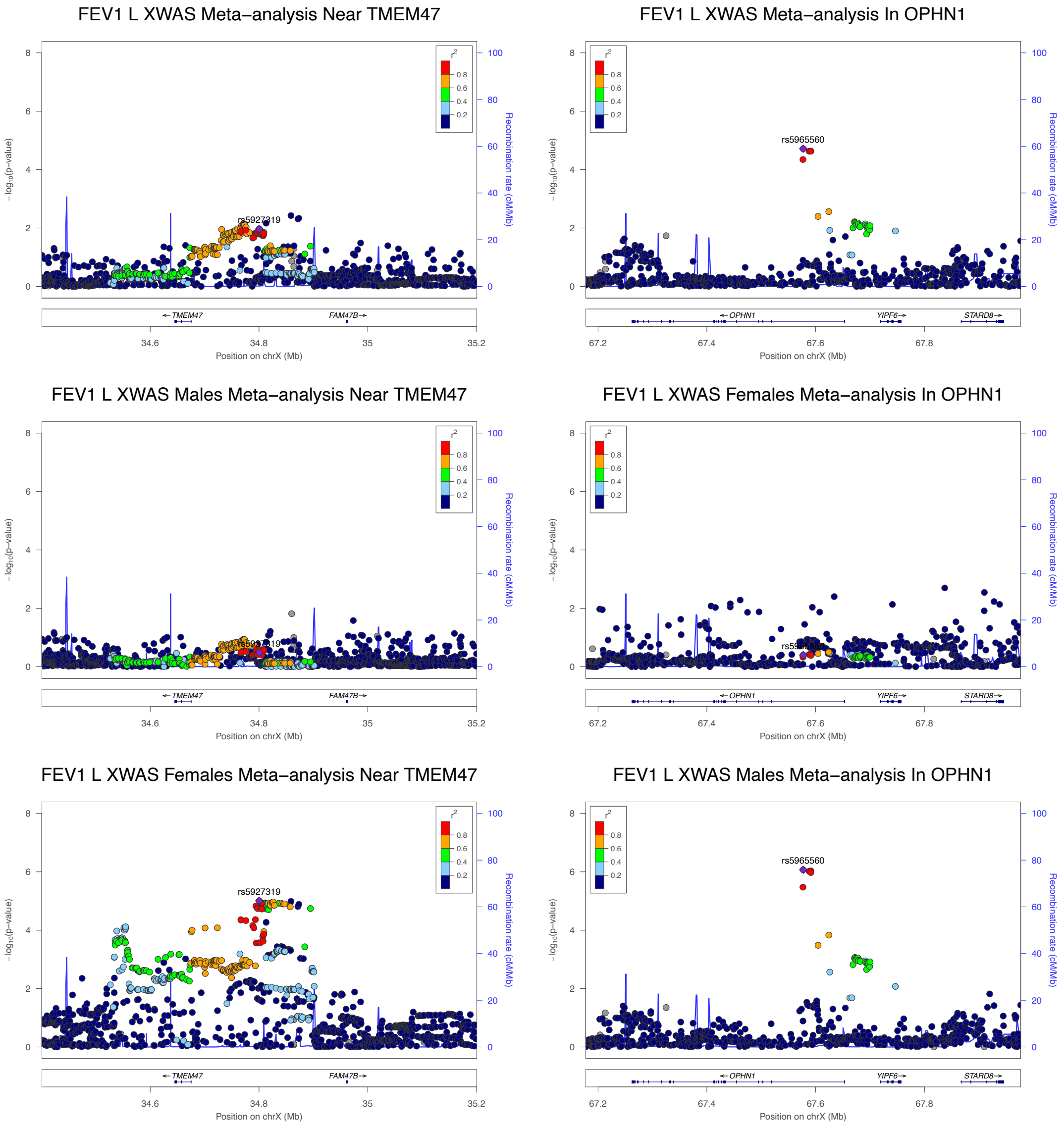
FEV1 L XWAS Meta−analysis Near TMEM47
FEV1 L XWAS Meta−analysis In OPHN1
FEV1 L XWAS Males Meta−analysis Near TMEM47
FEV1 L XWAS Females Meta−analysis In OPHN1
FEV1 L XWAS Females Meta−analysis Near TMEM47
FEV1 L XWAS Males Meta−analysis In OPHN1
rs5927319
rs5965560
r2
0.8
0.6
0.4
0.2
− log10(p−value)
Recombination rate (cM/Mb)
TMEM47
FAM47B
OPHN1
YIPF6
STARD8
34.6
34.8
35
35.2
67.2
67.4
67.6
67.8
Position on chrX (Mb)

FEV$_1$/FVC

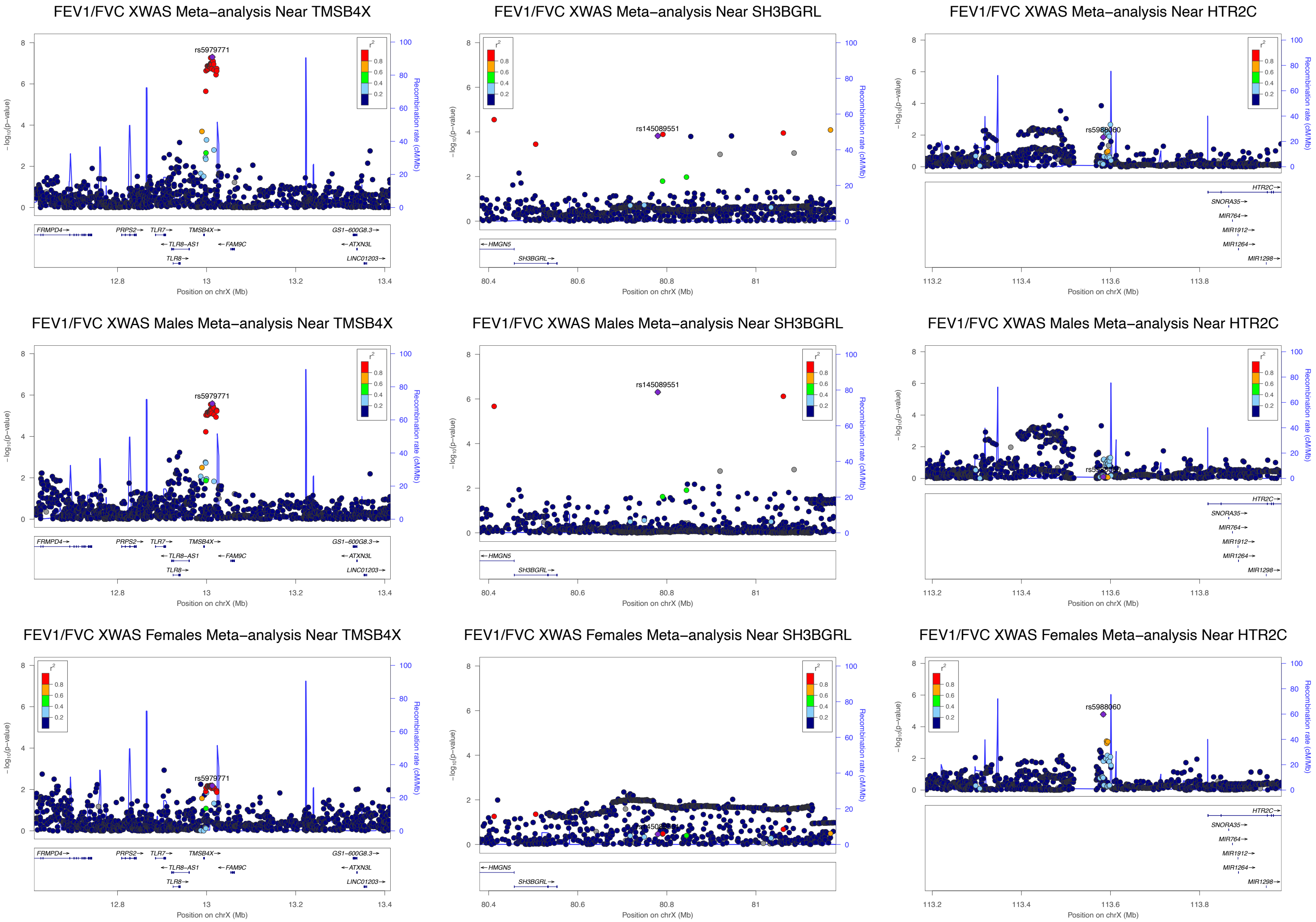

FEV1/FVC XWAS Meta–analysis Near TMSB4X
FEV1/FVC XWAS Meta–analysis Near SH3BGRL
FEV1/FVC XWAS Meta–analysis Near HTR2C
FEV1/FVC XWAS Males Meta–analysis Near TMSB4X
FEV1/FVC XWAS Males Meta–analysis Near SH3BGRL
FEV1/FVC XWAS Males Meta–analysis Near HTR2C
FEV1/FVC XWAS Females Meta–analysis Near TMSB4X
FEV1/FVC XWAS Females Meta–analysis Near SH3BGRL
FEV1/FVC XWAS Females Meta–analysis Near HTR2C
rs5979771
rs145089551
rs5988060
−log10(p−value)
Recombination rate (cM/Mb)
Position on chrX (Mb)
r2
FRMPD4
PRPS2
TLR7
TMSB4X
GS1−600G8.3
TLR8−AS1
FAM9C
ATXN3L
TLR8
LINC01203
HMGN5
SH3BGRL
HTR2C
SNORA35
MIR764
MIR1912
MIR1264
MIR1298

Emphysema

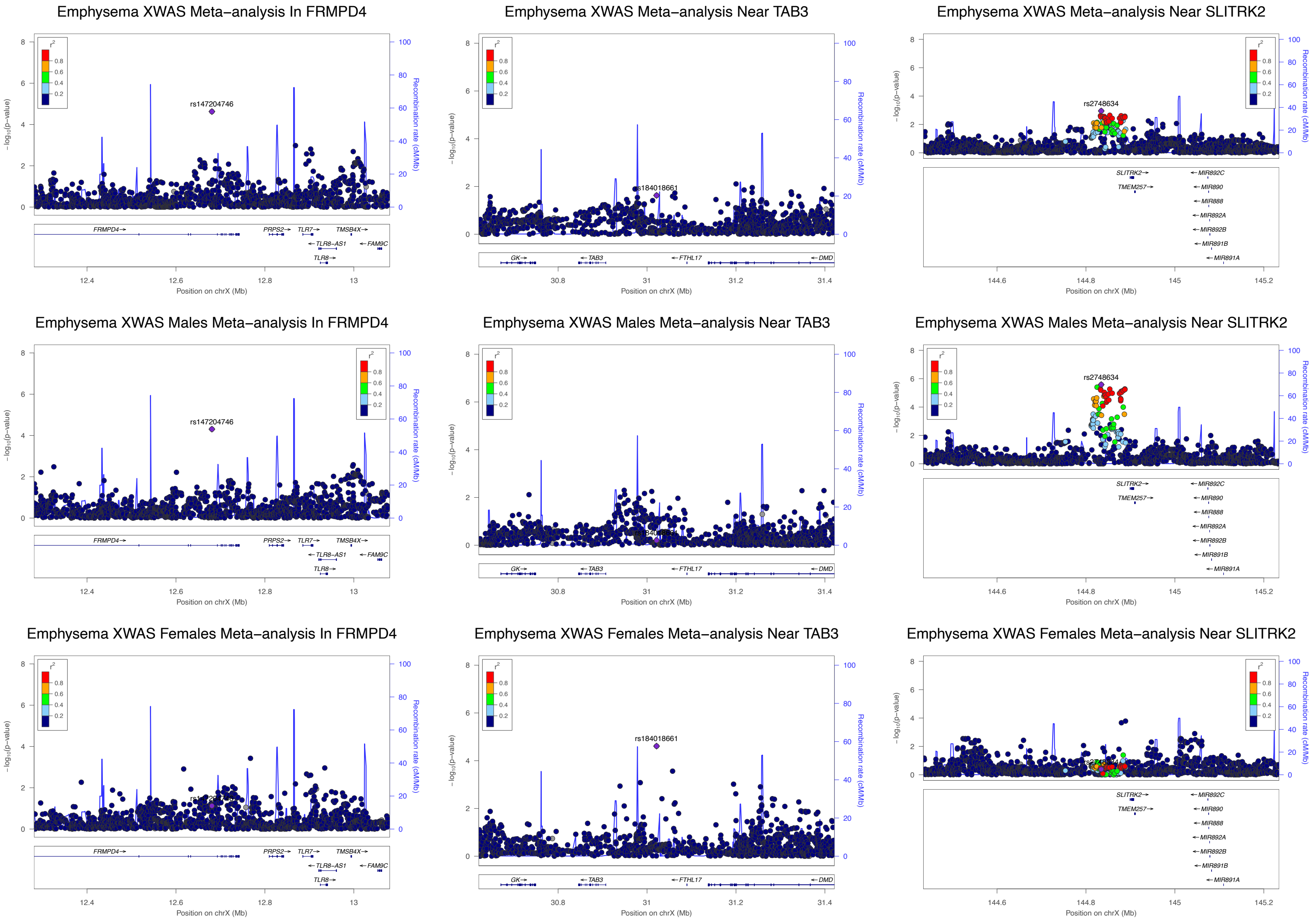

Supplement Figure 3 Locus plots for additional top suggested associations in meta-analysis among all subjects and in sex-stratified populations. Abbreviations: *COPD* chronic obstructive pulmonary disease; *XWAS* X chromosome association study; *FEV₁* Forced expiratory volume in one second; *L* liters; *FVC* forced vital capacity.

Supplement Figure 4: Meta-analysis Quantile-Quantile and Manhattan Plots

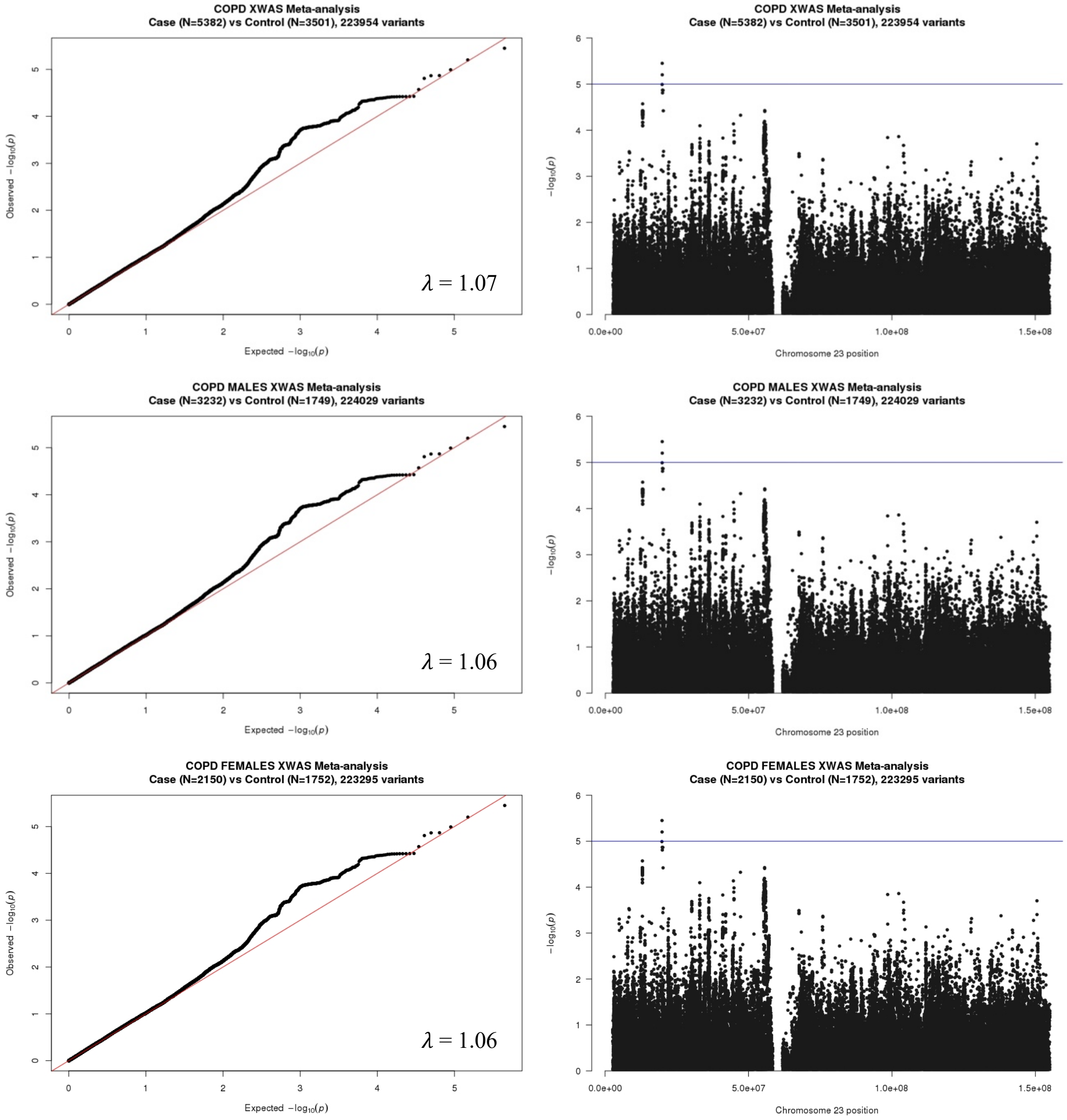

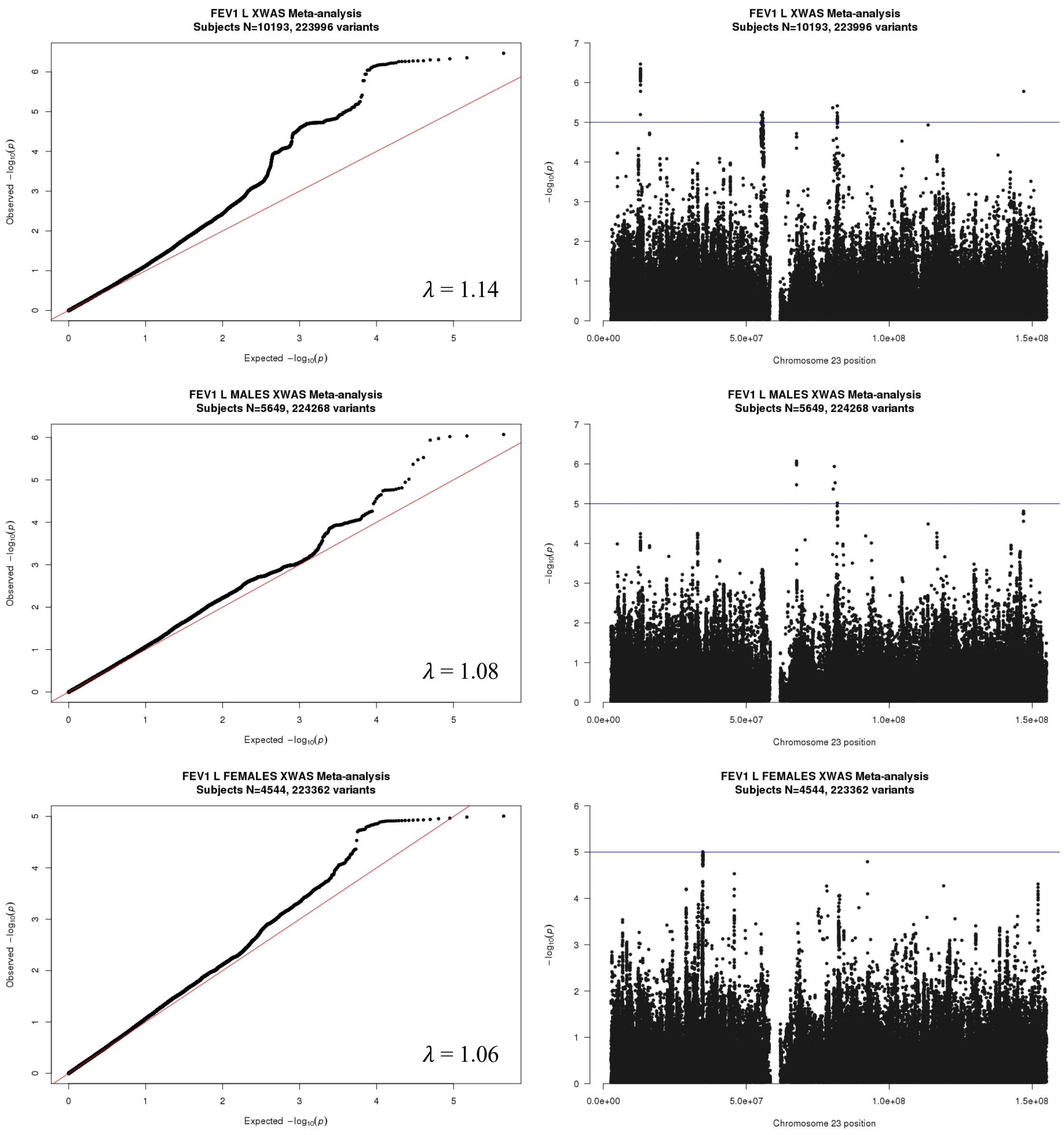
FEV1 L XWAS Meta-analysis
Subjects N=10193, 223996 variants
Observed −log10(p)
Expected −log10(p)
λ = 1.14
FEV1 L XWAS Meta-analysis
Subjects N=10193, 223996 variants
−log10(p)
Chromosome 23 position
FEV1 L MALES XWAS Meta-analysis
Subjects N=5649, 224268 variants
Observed −log10(p)
Expected −log10(p)
λ = 1.08
FEV1 L MALES XWAS Meta-analysis
Subjects N=5649, 224268 variants
−log10(p)
Chromosome 23 position
FEV1 L FEMALES XWAS Meta-analysis
Subjects N=4544, 223362 variants
Observed −log10(p)
Expected −log10(p)
λ = 1.06
FEV1 L FEMALES XWAS Meta-analysis
Subjects N=4544, 223362 variants
−log10(p)
Chromosome 23 position

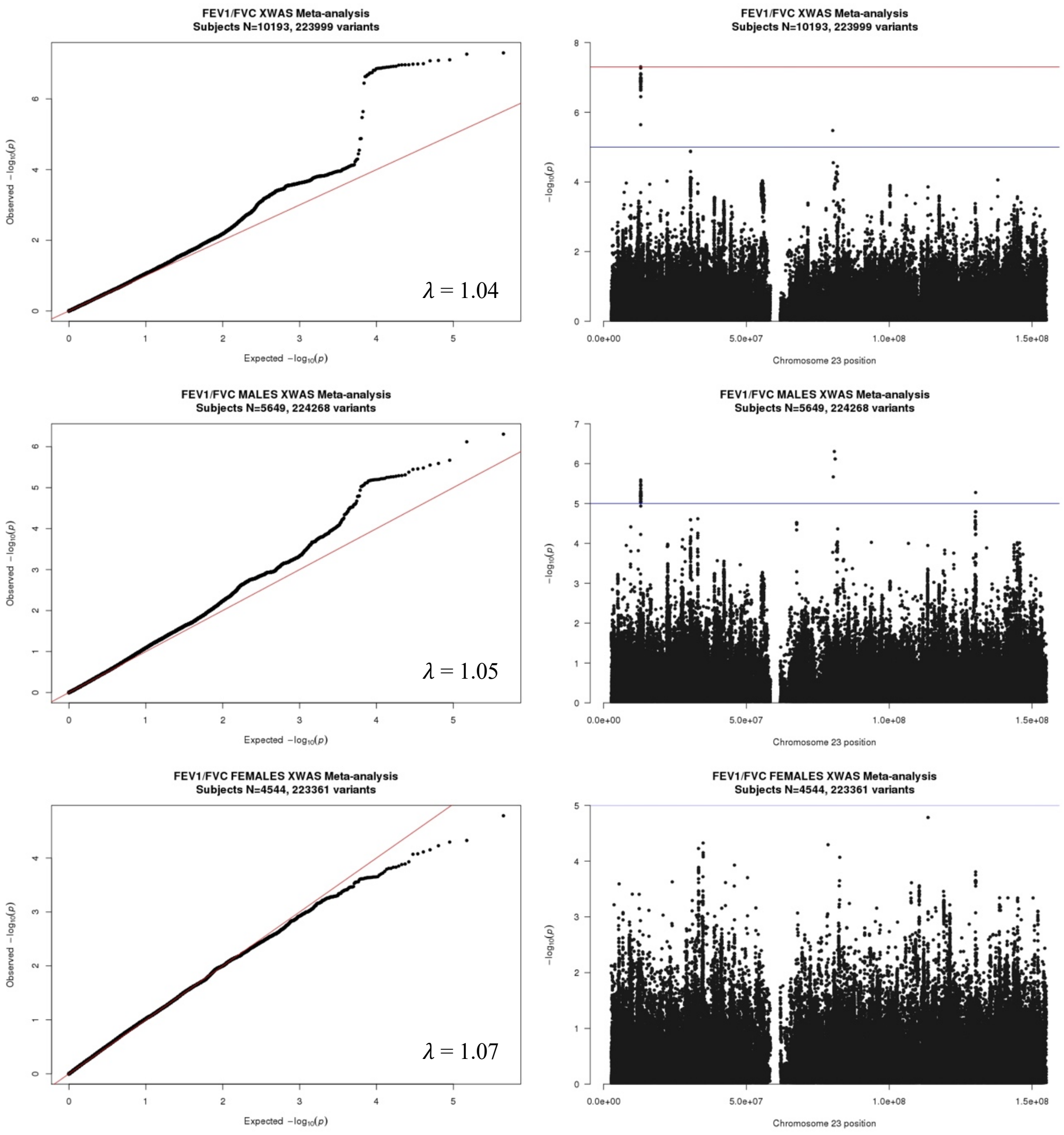
FEV1/FVC XWAS Meta-analysis
Subjects N=10193, 223999 variants
λ = 1.04
FEV1/FVC MALES XWAS Meta-analysis
Subjects N=5649, 224268 variants
λ = 1.05
FEV1/FVC FEMALES XWAS Meta-analysis
Subjects N=4544, 223361 variants
λ = 1.07
Observed −log10(p)
Expected −log10(p)
−log10(p)
Chromosome 23 position
0.0e+00
5.0e+07
1.0e+08
1.5e+08

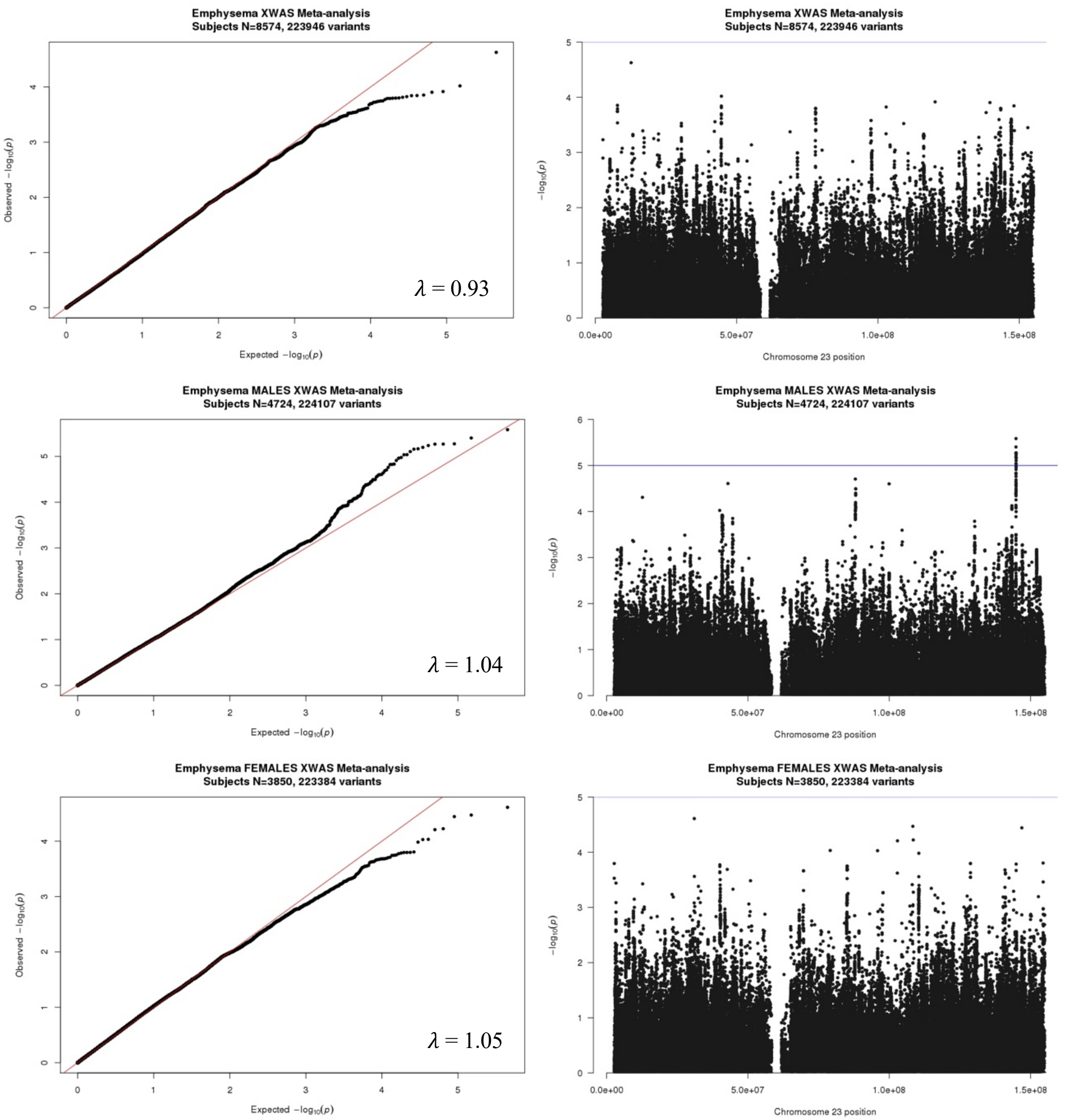

Supplement Figure 4 QQ and Manhattan plots for the twelve XWAS meta-analyses. Abbreviations: *COPD* chronic obstructive pulmonary disease; *XWAS* X chromosome association study; *FEV_1* Forced expiratory volume in one second; *L* liters; *FVC* forced vital capacity.

Supplement Figure 5: Sex differential edge weights connecting the transcription factor *POU3F4* in GTEx Lung Tissue

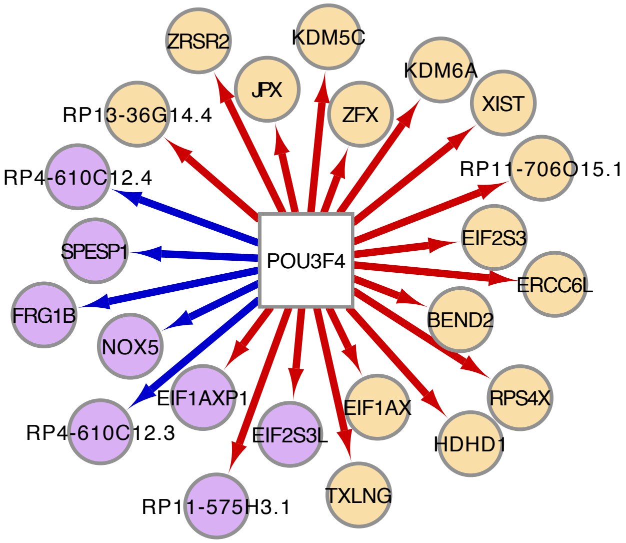

Supplement Figure 5 Sex differential edge weights connecting the transcription factor *POU3F4* to 23 target genes (FDR<0.05). Includes 18 female-biased edges (red), 5 male-biased edges (blue). Yellow nodes represent target genes in the X chromosome and purple notes in the autosome.
